# Aromatic Thioacetal-Bridged ROS-Responsive Nanoparticles as Novel Gene Delivery Vehicles

**DOI:** 10.3390/molecules23082061

**Published:** 2018-08-17

**Authors:** Guo-Qing Lin, Wen-Jing Yi, Qiang Liu, Xue-Jun Yang, Zhi-Gang Zhao

**Affiliations:** College of Chemistry and Environmental Protection Engineering, Southwest Minzu University, Chengdu 610041, China; lgq1012966356@163.com (G.-Q.L.); lqchem@163.com (Q.L.); yangxuejun@swun.edu.cn (X.-J.Y.)

**Keywords:** reactive oxygen species (ROS), stimuli responsive, gene delivery, cationic polymer, non-viral vectors

## Abstract

In this report, a series of polycations are designed and synthesized by conjugating reactive oxygen species (ROS)-responsive thioacetal-linkers to low molecular weight (LMW) polyethylenimine (PEI) via ring-opening polymerization. Their structure–activity relationships (SARs) as gene delivery vectors are systematically studied. Although the MWs of the target polymers are only ~9 KDa, they show good DNA binding ability. The formed polyplexes, which are stable toward serum but decomposed under ROS-conditions, have appropriate sizes (180~300 nm) and positive zeta-potentials (+35~50 mV). In vitro experiments reveal that these materials have low cytotoxicity, and higher transfection efficiency (TE) than controls. Furthermore, the title polymers exhibit excellent serum tolerance. With the present of 10% serum, the TE of the polymers even increases up to 10 times higher than 25 KDa PEI and 9 times higher than Lipofectamine 2000. The SAR studies also reveal that electron-withdrawing groups on the aromatic ring in **4a** may benefit to balance between the DNA condensation and release for efficient gene transfection.

## 1. Introduction

Gene therapy is considered to be one of the most promising approaches to treating various human genetic and acquired diseases, such as cancers, aging, diabetes, and cardiovascular diseases [1]. Safe and effective gene delivery vectors are a prerequisite for successful gene therapy [2,3]. Due to the high transfection efficiency (TE), viral carriers are a commonly used method of gene delivery. However, their immunogenicity, toxicity, limited DNA packaging capacity, and difficulty in large-scale production have impeded their development and clinic applications [4,5]. In attempt to overcome these barriers, a broad range of versatile and efficient non-viral gene vectors have been developed. Cationic polymers, as one class of non-viral gene vectors, have acquired increasing attention due to their easy preparation and modification in recent years [6,7,8]. 

Gene transfection with cationic polymers needs to overcome multiple delivery barriers, including the formation of polymers/DNA complexes (polyplexes) and their initial binding to the cell surface, endocytosis, endosomal escape, nuclear entry, and final expression [9,10,11]. “Golden standard” 25 KDa PEI is the most widely studied polymeric carrier, both in vitro and in vivo [12]. Unfortunately, high molecular weight (HMW) PEI remains cationic under physiological conditions and is not biodegradable, resulting in its well-known cytotoxicity, the difficult release of genes and low TE under serum conditions [13,14]. Compared to the HMW PEI, the LMW PEI illustrates much lower toxicity, yet almost no transfection activity [15]. Recently, plentiful approaches to obtaining high TE using covalently conjugated and non-conveniently assembled LMW PEI have been investigated [16,17]. One of the feasible approaches is to cross-link LMW PEI with biodegradable linkers (ester, ketal and disulfide etc.) [13,18,19] to form “smart” polymers that can eventually degrade into small molecules and simultaneously release complexed nucleic acids in the internal or external stimuli (pH, redox, temperature, light, and magnetic field etc.) [20,21,22,23]. 

Such endogenous stimuli that are drawing keen attention recently are reactive oxygen species (ROS), which include hydrogen peroxides (H_2_O_2_), hydroxyl radicals (·OH), peroxynitrites (ONOO^−^) and superoxides (O_2_^−^) [24,25,26,27]. Commonly, ROS are generated from mitochondrial electron transport as a byproduct, and they are used in cell signaling of normal cells or cause oxidative stress from overproduction [28]. On the other hand, a number of evidences reveal that chronically increased levels of ROS are closely associated with various pathological disorders including cancer, atherosclerosis, diabetes, infections, inflammatory diseases, where ROS levels can be 10- to 100-times higher than those of the normal levels [29,30,31]. Therefore, ROS can be considered as a target or an indicator for selectively delivering cargo molecules to diseased sites by targeting oxidative microenvironment at different levels. Some materials that can undergo phase transition or hydrolyze under oxidative conditions therefore have been developed to construct ROS-sensitive carriers. Alternatively, some oxidation-labile groups (thioether, thioketal [25,26,32], selenium-containing groups, oligoproline peptide and boronic acid esters etc.) are incorporated into polymers as ROS-responsive groups [33,34,35,36]. It was hypothesized that the ROS-responsive nanocarriers could facilitate the intracellular gene release and transfection expression inspired by the high level of intracellular ROS.

**Scheme 1:**
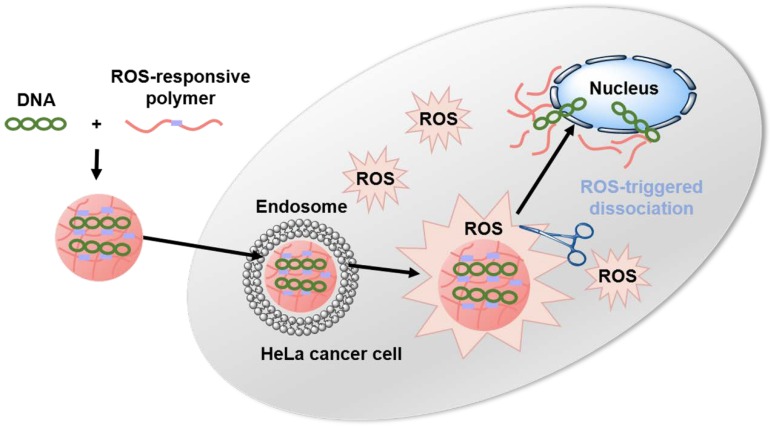
Illustration of intracellular delivery of plasmid DNA to the nucleus of HeLa cancer cell using ROS-responsive polymer based LWM PEI and thioacetal-linker units.

Based on the above issues, the aim of this study was to develop novel biodegradable polymeric nanocarriers based on LWM PEI and thioacetal-linker units, exploiting the high level of ROS in cancer cells (as shown in Scheme 1). In addition, rigid aromatic structure containing polymers showed increased TE was recently reported by our group [37,38,39]. The aromatic rings may help to improve the DNA condensation ability of cationic polymers. Besides, we also found diepoxide ring-opening polymerization is a practical synthetic method toward polymeric vectors with high biological compatibility by the newly formed hydroxyls and oxygen atoms similar to PEG structure [38,39,40,41,42]. Hence, we prepared this series of polycations through the epoxide ring-opening polymerization from bis-epoxides with various aromatic thioacetal groups. Then, their gene delivery properties (such as DNA condensation, cytocompatibility, in vitro TE) and structure-property correlations were systematically investigated.

## 2. Results and Discussion

### 2.1. Polymer Synthesis and Characterization

**Scheme 2:**
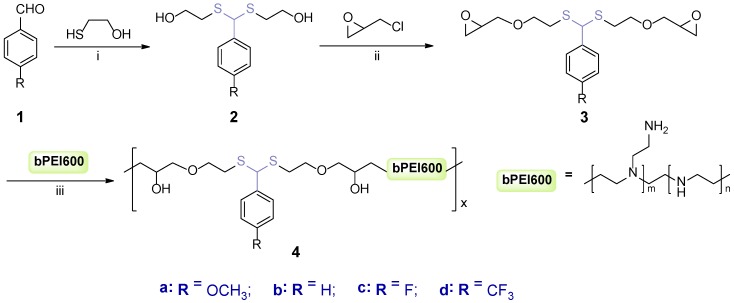
Synthetic route of the target polymers. (i) NiCl_2_·6H_2_O, rt. (ii) epichlorohydrin, Bu_4_NBr, NaOH, NaOH, H_2_O, 35 °C. (iii) EtOH, reflux.

The preparation route for the target polymers is shown in Scheme 2. Various aromatic aldehydes **1** reacted with 2-mercaptoethanol to give compound **2** in the presence of catalyst NiCl_2_·6H_2_O. Subsequently, diglycidyl ether **3** was prepared by the reaction of **2** with epichlorohydrin in the presence of NaOH and water with Bu_4_NBr as a phase transfer catalyst. The ring-opening polymerization was simply processed between equal molar amounts of diglycidyl ethers cross-linker **3** and PEI 600 Da in refluxed ethanol. The crude product was recrystallized for 3 times with anhydrous DCM and methanol to ensure their polydispersity. The obtained polymers showed significant water solubility, which was required for the subsequent studies. Moreover, four diglycidyl ether bridges with varied electron-effects (electron-donating and electron-withdrawing substituents) were introduced for helping us to elucidate the structure–activity relationships (SARs). These new compounds were characterized by ^1^H-NMR (400 MHz) using D_2_O as a solvent. Although the molar ratio of the substrates (bridge and PEI 600) for polymerization was 1:1, the integral ratio of the proton signals from the bridge (18H each) and PEI (56H each) revealed that the units of the bridge were more than the latter, indicating the formation of a reticular but not linear structure (Supplementary Materials). The molecular weights of target polymers **4** were measured by GPC, and results in Table 1 showed that the M_w_s were found to be in the range of 7–10 KDa and the PDIs (polymer dispersity index) were relatively high around 1.3~2. Because it is well-known that the higher molecular weight of polymers always comes with higher cytotoxicity [38], we hope our materials would have lower toxicity while maintaining high TE. 

### 2.2. Polymer Responds to Reactive Oxygen Species

With the polymer **4** in hand, we investigated its response to ROS under simulated conditions. In a typical experiment, polymer **4** was dissolved in D_2_O containing 100 mM H_2_O_2_ to trigger its degradation. When the polymer was incubated at 37 °C for different times, the disappearance of thioketal linkage peak (δ ≈ 5.20 ppm) was monitored by ^1^H-NMR spectroscopy (Figure S2, Supplementary Materials). The ^1^H-NMR spectrum confirmed that the thioketal linkages were efficiently cleaved by ROS, generating substituted benzaldehyde (δ ≈ 8.25 ppm) as a byproduct during the cleavage process.

### 2.3. Interaction with Plasmid DNA and Characterization of Polyplexes

**Figure 1:**
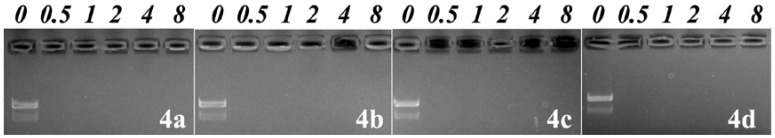
Agarose gel electrophoresis assays of polymer/DNA (pUC19) complexes at different *w*/*w* ratios. In each image, the first lane is the DNA control.

Condensation of negatively charged DNA into nano-sized particles is a prerequisite of great importance for well-designed polymeric vectors. The formation of a polyplex can reduce the electrostatic repulsion between DNA and the cell surface, and can protect DNA against enzymatic degradation by nucleases in cytoplasm or serum [43]. Agarose gel electrophoresis was used to evaluate the DNA binding ability between the polymer and DNA at various weight ratios (polymer/DNA, *w*/*w*), and results were shown in Figure 1. All 4 materials exhibited good DNA binding capacity, and full DNA retardation could be observed at a relatively low dosage from the *w*/*w* ratio of ~0.5. The structure of aromatic substituent seemed to have little effect on their DNA retardation ability. However, polymers could not efficiently complex with DNA even at a high *w*/*w* ratio of 8 after exposed to ROS (Figure S3, Supplementary Materials). This finding implied that polymer **4** was decomposed following incubation with H_2_O_2_.

**Figure 2:**
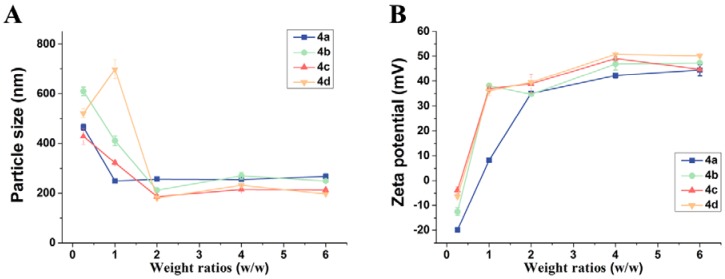
Particle size (**A**) and zeta-potential (**B**) of polymer/DNA polyplexes obtained at various *w*/*w* ratios by DLS. Data are represented by mean ± standard deviation (S.D.) (*n* = 3).

Proper particle size and positive surface charge are essential for efficient gene delivery. It was reported that complexes within the size range of 50 to several hundred nanometers would receive easier endocytosis [44]. Dynamic light scattering (DLS) measurements showed that the particle sizes of **4**/DNA polyplexes changed dramatically at relatively low *w*/*w* ratios (0.5–1), and became constant at 180~300 nm with the increase of *w*/*w* ratio (Figure 2A). The larger particles formed at the *w*/*w* ratio of 0.5–1 might be attributed to their neutral zeta-potential, which led to the minimized repulsion and resulting aggregation. Meanwhile, we also examined the possible changes of particle sizes of **4a**/DNA at a *w*/*w* ratio of 2 in the presence of 10% serum. Results suggested that the particle sizes of polyplexes were still stable at ~250 nm and seldom altered, indicating that no apparent aggregation occurred (Figure S1, Supplementary Materials). Zeta potential shown in Figure 2B indicated the surface charge rose along with the increase of *w*/*w* ratios, and reached about +35~50 mV above a *w*/*w* ratio of 2. Interestingly, the polyplexes with electron-donating substitute revealed lower zeta potential than the electron-withdrawing ones, which might be due to the positive charge screening by the electron-rich effect. Generally, the positively charged complexes can interact with the negatively charged cellular membranes, leading to more efficient cellular uptake [40]. 

### 2.4. In Vitro Gene Transfection

**Figure 3:**
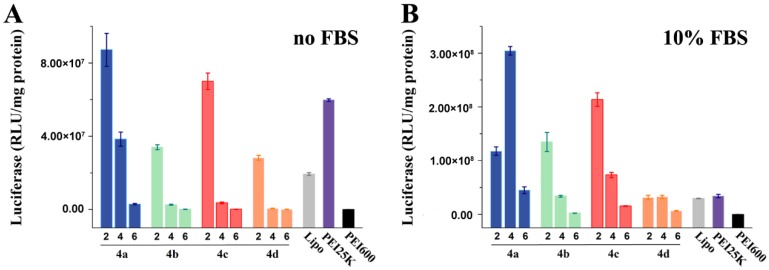
Luciferase gene expression transfected by polyplexes at different weight ratios: 2, 4, and 6 in comparison with Lipofectamine 2000, PEI 600 (*w*/*w* = 6, N/P = 28.7) and 25 KDa PEI (*w*/*w* = 1.4, N/P = 10) in human human cervix carcinoma cell lines (HeLa) in the absence of serum (**A**) and in the presence of 10% serum (**B**). Data are represented by mean ± S.D. (*n* = 3).

The in vitro TE of the new polyplexes was evaluated in HeLa cells with high ROS levels by using pGL3 plasmid as a luciferase reporter gene. Figure 3A shows the relative TEs at various *w*/*w* ratios in a serum-free medium, and Lipofectamine 2000, 25 KDa PEI and PEI 600 were used as controls. All the polymers at *w*/*w* ratios of 2 and 4 gave comparable TEs to commercial reagent Lipofectamine 2000. To our delight, polymer **4a** gave the highest TE, which was nearly 4.5 times higher TE than Lipofectamine 2000 and 1.5 times higher TE than 25 KDa PEI at a *w*/*w* ratio of 2. The electron-withdrawing groups on the aromatic ring seemed to play an important role in the transfection process. We speculate that the lower zeta potentials caused by the electron-rich effect in **4a** might benefit the dissociation of the polyplexes, leading to better release of DNA in the cytosol [40].

In general, the interaction between negatively charged serum proteins and polycationic gene vector would inhibit efficient gene delivery [45]. Previous results in DLS experiment showed good serum tolerance of our new material. The transfection ability of the polymers in the presence of serum was also estimated by the same method. As shown in Figure 3B, it was found that the relative TE was even higher for all synthesized polymers in the presence of 10% of serum, indicating that these polyplexes were quite stable and were seldom affected by the serum. Especially **4a** polyplexes, demonstrated approximately 10 times higher TE than Lipofectamine 2000 and 9 times higher than 25 KDa PEI, respectively. Besides the aromatic group, the hydroxyls formed from ring-opening polymerization would also contribute to the excellent serum tolerance [38,41].

**Figure 4:**
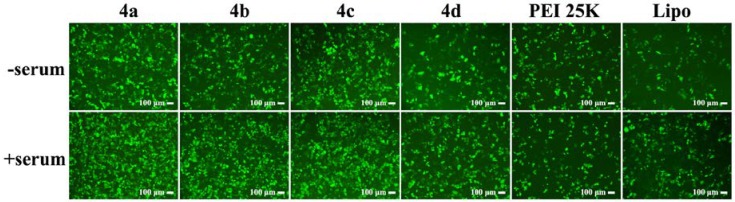
Fluorescence microscope image of pEGFP-transfected HeLa cells at optimal weight ratios in the absence of serum (2 for **4a**–**d**) or in the presence of 10% FBS (4 for **4a** and **4d**, 2 for **4b** and **4c**).

To directly visualize the infected cells expressing green fluorescent protein reporter gene pEGFP-Nl, enhanced green fluorescent protein expression in HeLa cells was observed by an inverted fluorescent microscope. The *w*/*w* ratios were used according to the optimal results from luciferase assays, and the images are shown in Figure 4. Similar to the results in luciferase assay, polymer **4a** gave the strongest green fluorescence among the four polymers, and the density of expressed GFP was also higher than that involving control PEI 25 KDa and Lipofectamine 2000. Serum seemed to have no negative effect on the transfection mediated by **4**; however, PEI 25 KDa-participated transfection was inhibited by serum on account of high positive charges. All results further demonstrated the good serum tolerance of our new vectors.

### 2.5. Cytotoxicity 

**Figure 5:**
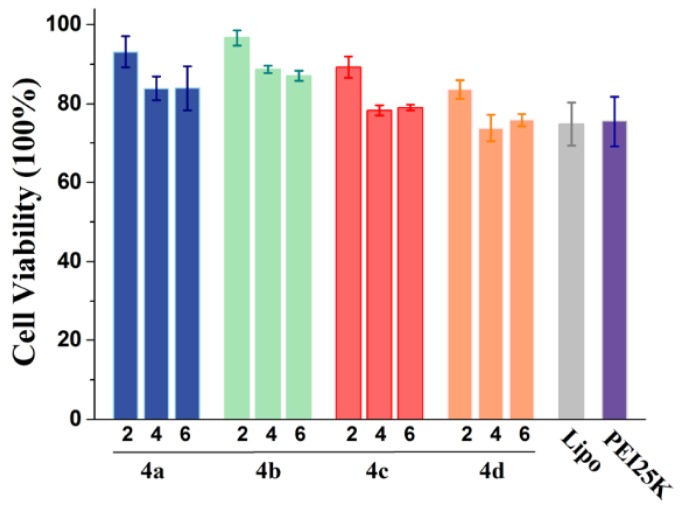
Cytotoxicity of polyplexes at different *w*/*w* ratios toward HeLa cells. Lipofectamine 2000 and PEI 25 KDa were used as controls (*w*/*w* = 1.4, N/P = 10). Data are represented by mean ± S.D. (*n* = 3).

Successful gene transfection requires efficient transfection and minimized cytotoxicity. However, most of the polymeric are caught in the dilemma: either high TE associated with severe cytotoxicity, or low TE with better compatibility [26]. These new polycations have molecular weight around 9 KDa, and previous experiments exhibited that although their molecular weights are relatively low, they could completely condense DNA even at low *w*/*w* ratios (~0.5). We hope their small molecular weights may result in low toxicity. The cytotoxicities of the polyplexes were studied and compared with PEI 25 KDa and Lipofectamine 2000 in HeLa cells via CCK-8 assays. It should be noted that the *w*/*w* ratio for CCK-8 assay was the same as that used in the transfection experiment. As shown in Figure 5, the cell viabilities of the studied materials were higher than those of 25 KDa PEI and Lipofectamine 2000 even at high *w*/*w* ratios in most cases, suggesting that these polymers had better biocompatibility. Comparing to the structure of bPEI, we believe that the lower cytotoxicity may be attributed to the newly formed hydroxyl groups and biodegradable thioacetal, which can benefit the biocompatibility of the polymeric materials used for gene delivery. It was also proved that the ring-opening polymerization from bis-epoxides is an effective synthetic approach toward materials with low cytotoxicity.

## 3. Experimental Section

### 3.1. Materials and Methods

Anhydrous MeOH, EtOH and dichloromethane (DCM) were distilled immediately before use after drying and purifying by using standard methods. 2,2′-((4-methoxyphenyl)methylene)bis(sulfanediyl))diethanol (**2a**), 2,2′-((phenylmethylene)bis(sulfanediyl))diethanol (**2b**) and 2,2′-((4-fluorophenyl)methylene)bis(sulfanediyl))diethanol (**2c**) were prepared according to the literature [46]. 0.6 KDa and 25 KDa branched polyethylenimine (PEI) (99%, Sigma-Aldrich, St. Louis, MO, USA), Dulbecco’s Modified Eagle’s Medium (DMEM) and fetal bovine serum (FBS) (Invitrogen Corp., Carlsbad, CA, USA), MicroBCA protein assay kit (Pierce, Appleton, WI, USA), Luciferase assay kit (Promega, Madison, WI, USA), Endotoxin free plasmid purification kit (TIANGEN, Beijing, China) were used as received. Luciferase (pGL-3) and green fluorescent protein (pEGFP-N1) plasmid DNA were purchased from Promega and Clontech. The supercoiled plasmid DNA (pUC-19) used in agarose-gel assay was purchased from Takara (Dalian, China). HeLa cells (human cervix carcinoma cell lines) were purchased from Shanghai Institute of Biochemistry and Cell Biology, Chinese Academy of Sciences. All the other chemicals reagents and solvents of analytical grade, if not specified, were used as received from commercial sources without further purification.

The ^1^H-NMR spectra were recorded at 400 MHz and ^13^C spectra at 100 MHz on a Varian INOVA-400 spectrometer (Varian, Palo Alto, CA, USA). The high-resolution mass spectrometry (HRMS) experiments were performed using a Finnigan LCQDECA (Thermo Scientific, Waltham, MA, USA) and a Bruker Daltonics BioTOF mass spectrometer (Bruker Daltonics Inc., Billerica, MA, USA). The M_w_ and polydispersity index (PDI) of the target polymers were determined by gel permeation chromatography (GPC) system, which consisted of Shodex columns OHPAK KB-803, Waters 515 pump and Waters 2410 Refractive Index Detector. 0.5 mol·L^−1^ HAc/NaAc buffer was passed through a filter and used as the mobile phase, at a flow rate of 0.5 mL·min^−1^. M_w_s were calculated against PEG standards of M_n_ ranging from 900 to 80,000.

### 3.2. Synthesis of Title Polymers

#### 3.2.1. Preparation of 2,2′-((4-(Trifluoromethyl)phenyl)methylene)bis(sulfanediyl))diethanol (Compound **2d**)

4-(Trifluoromethyl)benzaldehyde (**1d**, 20 mmol, 3.48 g) was mixed with 2-mercaptoethanol (40 mmol, 3.12 g). After addition of the catalyst NiCl_2_·6H_2_O (0.1 mmol, 23.8 mg), the solution was stirred at room temperature for 1 h, and then 10 mL DCM was added to separate out the catalyst by filtering off. The solvent was concentrated to afford a white solid, which was purified by column chromatography on a silica gel (DCM:MeOH = 40:1, *v*/*v*) to give compound **2d** as a white solid. Yield: 77.6%. ^1^H-NMR (400 MHz, CDCl_3_) δ (ppm) 7.69–7.48 (m, 4H, Ph*H*), 5.18 (s, 1H, -SC*H*S-), 3.78 (t, *J* = 5.8 Hz, 4H, -C*H*_2_OH), 2.96–2.66 (m, 4H, -C*H*_2_CH_2_OH). ^13^C-NMR (100 MHz, CDCl_3_) δ (ppm) 144.16, 128.10, 125.77, 125.73, 125.70, 125.66, 61.57, 52.57, 35.32. HRMS (ESI): *m*/*z* calcd. for C_12_H_15_F_3_O_2_S_2_ (M+) 312.0466; found = 335.0358 ([M + Na]^+^, 100).

#### 3.2.2. Preparation of Diglycidyl Ether Linkers **3**

Compounds **3** were obtained using the method previously reported [37,40]. Briefly, compound **2** (0.02 mol), epichlorohydrin (4.67 mL, 0.06 mol), NaOH (2.4 g, 0.06 mol), tetrabutylammonium chloride (0.3224 g, 0.001 mol) and pure water (1 mL, 0.056 mol) were mixed at room temperature and stirred at 35 °C overnight. The solid residue was separated out by filtering off and washed with DCM. The combined solvent was concentrated, and the residue was purified by a silica gel column chromatography to give the compound **3**. Analytical data for new compounds 3**a**–**d** were:

**3a**: white solid, yield: 31.2%. Eluent: PE/EA = 3:1 (*v*/*v*). ^1^H-NMR (400 MHz, CDCl_3_): δ (ppm) 7.39–6.84 (m, 4H, Ph*H*), 5.12 (s, 1H, -SC*H*S-), 3.81–3.78 (m, 3H, -OC*H*_3_), 3.76–3.33 (m, 8H, -C*H*_2_OC*H*_2_-), 3.13 (m, 2H, ring C*H*), 2.85–2.77 (m, 4H, -SC*H*_2_), 2.73–2.59 (m, 4H, ring C*H*_2_). ^13^C-NMR (100 MHz, CDCl_3_): δ (ppm) 159.18, 132.14, 128.92, 113.84, 71.50, 70.96, 55.25, 53.26, 50.72, 44.11, 31.73. HRMS (ESI): *m*/*z* calcd. for C_18_H_26_O_5_S_2_ (M+) 386.1222; found = 409.1117 ([M + Na]^+^, 100).

**3b**: white solid, yield: 33.6%. Eluent: PE/EA = 3:1 (*v*/*v*). ^1^H-NMR (400 MHz, CDCl_3_): δ (ppm) 7.45–7.25 (m, 4H, Ph*H*), 5.12 (s, 1H, -SC*H*S-), 3.76–3.35 (m, 8H, -C*H*_2_OC*H*_2_-), 3.14–3.12 (m, 2H, ring C*H*), 2.85–2.78 (m, 4H, -SC*H*_2_), 2.75–2.60 (m, 4H, ring C*H*_2_). ^13^C-NMR (100 MHz, CDCl_3_): δ (ppm) 140.20, 128.55, 127.97, 127.73, 71.52, 70.96, 70.93, 53.84, 50.74, 44.15, 31.74. HRMS (ESI): *m*/*z* calcd. for C_17_H_24_O_4_S_2_ (M+) 356.1116; found = 379.1010 ([M + Na]^+^, 100).

**3c**: pale-yellow oil, yield: 28.6%. Eluent: PE/EA = 2:1 (*v*/*v*). ^1^H-NMR (400 MHz, CDCl_3_): δ (ppm) 7.50–7.00 (m, 4H, Ph*H*), 5.17 (s, 1H, -SC*H*S-), 3.79–3.33 (m, 8H, -C*H*_2_OC*H*_2_-), 3.19–3.10 (m, 2H, ring C*H*), 2.90–2.76 (m, 4H, -SC*H*_2_), 2.74–2.61 (m, 4H, ring C*H*_2_). ^13^C-NMR (100 MHz, CDCl_3_): δ (ppm) 163.39, 160.94, 136.16, 136.13, 129.52, 129.44, 115.50, 115.29, 71.56, 71.02, 53.08, 50.74, 50.73,44.09, 31.82, 30.90. HRMS (ESI): *m*/*z* calcd. for C_17_H_23_FO_4_S_2_ (M+) 374.1022; found = 397.0919 ([M + Na]^+^, 100).

**3d**: pale-yellow oil, yield: 21.3%. Eluent: PE/EA = 2:1 (*v*/*v*). ^1^H-NMR (400 MHz, CDCl_3_): δ (ppm) 7.62–7.57 (m, 4H, Ph*H*), 5.24 (s, 1H, -SC*H*S-), 3.82–3.29 (m, 8H, -C*H*_2_OC*H*_2_-), 3.17–3.10 (m, 2H, ring C*H*), 2.89–2.77 (m, 4H, -SC*H*_2_), 2.74–2.57 (m, 4H, ring C*H*_2_). ^13^C-NMR (100 MHz, CDCl_3_): δ (ppm) 144.52, 128.31, 128.20, 125.57, 125.53, 73.41, 72.45, 72.44, 72.20, 71.62, 71.08, 71.06, 69.51, 53.30, 50.77, 50.76, 45.78, 44.09, 31.88, 30.86. HRMS (ESI): *m*/*z* calcd. for C_18_H_23_F_3_O_4_S_2_ (M+) 424.0990; found = 447.0890 ([M + Na]^+^, 100).

#### 3.2.3. Synthesis and Characterization of Target Polymers **4**

In a typical polymerization procedure, PEI 600 Da (0.5 mmol) and diglycidyl ether linkers **3** (0.5 mmol) were dissolved with 1 mL of anhydrous EtOH in a container. Under the protection of nitrogen (N_2_), the reaction mixture was stirred at 80 °C for 72 h. The resulting crude product was recrystallized from anhydrous DCM/methanol (*v*/*v* = 1/1). The recrystallized oily precipitate was isolated and dried until a constant weight was achieved. The molecular weights of compounds **4** were measured by GPC.

**4a**: Yield: 35.6%. ^1^H-NMR (400 MHz, D_2_O): δ (ppm) 7.55–6.80 (m, 4H, Ph*H*), 5.18 (s, 1H, -SC*H*S-), 3.98–2.20 (m, 33H, -SC*H*_2_C*H*_2_O-, -OC*H*_2_C*H*(OH)C*H*_2_-, -NHC*H*_2_C*H*_2_-). GPC: M_w_ = 9871, PDI = 1.85.

**4b**: Yield: 37.1%. ^1^H-NMR (400 MHz, D_2_O): δ (ppm) 7.63–7.00 (m, 5H, Ph*H*), 5.17 (s, 1H, -SC*H*S-), 4.08–1.94 (m, 42H, -SC*H*_2_C*H*_2_O-, -OC*H*_2_C*H*(OH)C*H*_2_-, -NHC*H*_2_C*H*_2_-). GPC: M_w_ = 10,916, PDI = 1.97.

**4b**: Yield: 29.8%. ^1^H-NMR (400 MHz, D_2_O): δ (ppm) 7.45–6.64 (m, 4H, Ph*H*), 5.13 (s, 1H, -SC*H*S-), 4.11–2.01 (m, 35H, -SC*H*_2_C*H*_2_O-, -OC*H*_2_C*H*(OH)C*H*_2_-, -NHC*H*_2_C*H*_2_-). GPC: M_w_ = 7123, PDI = 1.70.

**4b**: Yield: 31.2%. ^1^H-NMR (400 MHz, D_2_O): δ (ppm) 7.77–7.18 (m, 4H, Ph*H*), 5.21 (s, 1H, -SC*H*S-), 4.02–2.05 (m, 38H, -SC*H*_2_C*H*_2_O-, -OC*H*_2_C*H*(OH)C*H*_2_-, -NHC*H*_2_C*H*_2_-). GPC: M_w_ = 8209 PDI = 1.40.

### 3.3. Reactive Oxygen Species-Triggered Degradation of Thioketal Linkages

The degradation of thioketal linkages in polymer **4** under ROS conditions was investigated by incubating **4** (5 mg) with 100 mM of H_2_O_2_ in 0.6 mL of D_2_O at 37 °C for various periods of time. The disappearance of the thioketal linkage peak (δ ~ 5.20 ppm) was analyzed by using ^1^H-NMR.

### 3.4. Agarose Gel Retardation

pUC-19 (5 μL of 0.025 mg/mL in H_2_O) was mixed with the required amount of polymer to obtain the varied *w*/*w* ratios in H_2_O (a total volume of 10 μL). After an incubation time of 30 min at 37 °C, the polyplex solution was loaded onto an agarose gel (0.7% *w*/*v* agarose gel containing GelRed^TM^) and electrophoresed at 120 V for 40 min. A Vilber Lourmat imaging system was utilized to image the agarose gels [36]. 

To investigate the polymer stability under ROS conditions, the polymer solution (1 mg/mL) was treated with 100 mM H_2_O_2_ for 48 h at 37 °C. Subsequently, 0.125 μg pUC-19 and the desired amount of polymers at vary *w*/*w* ratios were mixed in 10 μL of DI for 0.5 h. The samples were run at the same electrophoresis condition as described above.

### 3.5. Particle Size and Zeta Potential Measurements

A Malvern Instruments Zetasizer Nano-ZS (ZEN3600, Malvern Instruments Ltd., Worcestershire, UK) was used to measure the complexes size and zeta potential. Polymer/DNA complexes were prepared at varying *w*/*w* ratios as described above. Samples were then diluted up to 1 mL using purity water before being measured [36]. In the experiments with serum, FBS (10% final concentrations) was added to the formulated polyplexes and mixed for 30 s. The particle sizes of the polyplexes were determined using Dynamic light scattering (DLS). Data were shown as mean ± standard deviation (S.D.) based on three independent measurements.

### 3.6. Reactive Oxygen Species-Responsive of Polyplexes

The ROS-responsive tests were conducted by mixing the complexes (**4a**/DNA at *w*/*w* of 2) with 100 μM H_2_O_2_ (final concentrations) at 37 °C for 1 days. After 12 and 24 h, the polyplexes were measured through DLS to obtain particle sizes and size distributions.

### 3.7. In Vitro Transfection Experiments

Gene transfection of a series of complexes was investigated in HeLa cells using two different plasmids encoding for green fluorescence protein (pEGFP-N1) and luciferase (pGL-3). Cells were seeded at a density of 7 × 10^4^ cells per well in 24-well plates and incubated for 24 h to achieve ~70–80% confluency. The culture medium was replaced with FBS free or 10% FBS containing DMEM medium before transfection. Polymer/DNA (1 μg) complexes were added to each well and incubated at 37 °C. Control reagents 25 KDa PEI (at a *w*/*w* ratio of 1.4, N/P = 10) [4,14,17] and Lipofectamine 2000 were formulated with DNA based upon their recommended protocols. After 4 h of incubation, the transfection medium was replaced with fresh complete medium containing 10% FBS and further incubated for 20 h. The images of GFP expressed cells were taken with an inverted fluorescence microscope (Nikon Eclipse TE 2000E, Nikon, Tokyo, Japan).

For the luciferase expression, cells were washed twice with PBS (pH 7.4) and lysed using 100 μL 1× Lysis reporter buffer. The luciferase activity was quantified using the luciferase assay reagent (Promega). The total protein content of the cell lysate was analyzed by BCA protein assay. The gene TE was expressed as the relative fluorescence intensity per mg protein (RLU/mg protein). All transfection experiments were performed as replicates of three [17].

### 3.8. Cytotoxicity Assay

The cytotoxicity studies were performed using Cell Counting Kit-8 (CCK-8) assay. HeLa cells were seeded in 96-well plates at a density of 8000 cells per well and allowed to adhere for 24 h. Complexes prepared at varied *w*/*w* ratios (2, 4, 6 corresponding to final concentrations were 4, 8, 12 μg/mL) were added to the FBS free growth medium and left to incubated for 4 h. Following incubation, the wells were replaced with fresh complete medium and incubated for a further 24 h. Subsequently, 10 μL of CCK-8 was then added to each well and the cells were incubated for 1 h at 37 °C. After that, the aborbance was readed using enzyme-linked immunosorbent assay (ELISA) plate reader (Model 680, Bio-Rad, Hercules, CA, USA) at a wavelength of 490 nm. The relative cell viability (%) related to untreated control cells was calculated by test/control × 100 [42]. Values were given as mean of triplicates ± S.D.

### 3.9. Statistical Analysis

Results are summarized as mean ± S.D., and statistical significance of differences was evaluated by variance analysis (ANOVA): *p* values smaller than 0.05 were considered to be significant [39]. 

## 4. Conclusions

In summary, we have successfully synthesized a series of ROS-responsive materials by the ring-opening polymerization between PEI 600 and diepoxide containing stimuli-responsive thioacetal groups. Four diglycidyl ethers with various electron-effects were employed in the side chain structure to facilitate the study of the SAR. These materials could efficiently condense DNA into stable nanoparticles with proper sizes and zeta-potentials. Besides, the thioketal linkages in polymers could also be dissociated under ROS-rich conditions for efficient gene delivery. Compared with 25 KDa PEI and Lipofectamine 2000, these polymers showed much higher TE in HeLa cells, especially polymer **4a** with electron-withdrawing substituent on the aromatic ring. More importantly, unlike PEI, serum had little negative effect on the transfection by these materials, and their TEs were even increased with the presence of 10% serum. The better serum tolerance of the polymers might come from their evenly distributed hydroxyl groups. Moreover, the cationic polymers exhibited good cell viabilities. The collective data suggested such polymer could be a promising target nanocarrier exploiting the high levels of intracellular ROS in cancer cells. Further modifications of the polymer structure and in-depth mechanism studies are now in progress.

## Figures and Tables

**Table 1:** Molecular weights and polydispersities of the target polymers.

Polymers	M_w_ (Da)	PDI
**4a**	9871	1.85
**4b**	10,916	1.97
**4c**	7123	1.70
**4d**	8209	1.40

## Supplementary Materials

The following are available online. Figure S1–S3 and Table S1. Additionally, ^1^H, ^19^F and ^13^C-NMR and GPC data for the compounds are provided.
