# Supplementary material for: Aromatic Thioacetal-Bridged ROS-Responsive Nanoparticles as Novel Gene Delivery Vehicles
Source: Molecules. 2018 Aug 17;23(8):2061. doi: 10.3390/molecules23082061 (PMC6225261; doi:10.3390/molecules23082061)
Supplement: Supplementary file 1 [file molecules-23-02061-s001.pdf]

Supplementary Information

Aromatic thioacetal -Bridged ROS-Responsive Nanoparticles as Novel Gene Delivery Vehicles

Guo-Qing Lin, Wen-Jing Yi *, Qiang Liu, Xue-Jun Yang and Zhi-Gang Zhao*

College of Chemistry and Environmental Protection Engineering, Southwest Minzu University, Chengdu 610041, P.R. China

**Corresponding authors: Wen-Jing Yi, ywjchem@163.com;*

Zhi-Gang Zhao, zzg63129@163.com

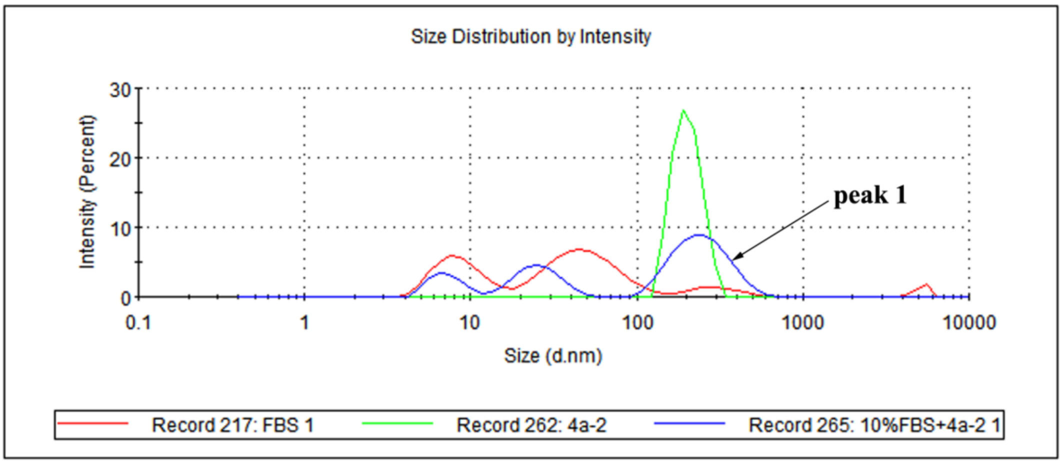

Fig. S1 The particle diameter obtained by DLS.
Green line: the dimeter of **4a**/DNA complexes at w/w ratio of 2 without FBS;
red line: the diameter of 10% FBS;
blue line: the diameter of **4a**/DNA complexes at w/w ratio of 2 in presence of 10% FBS.

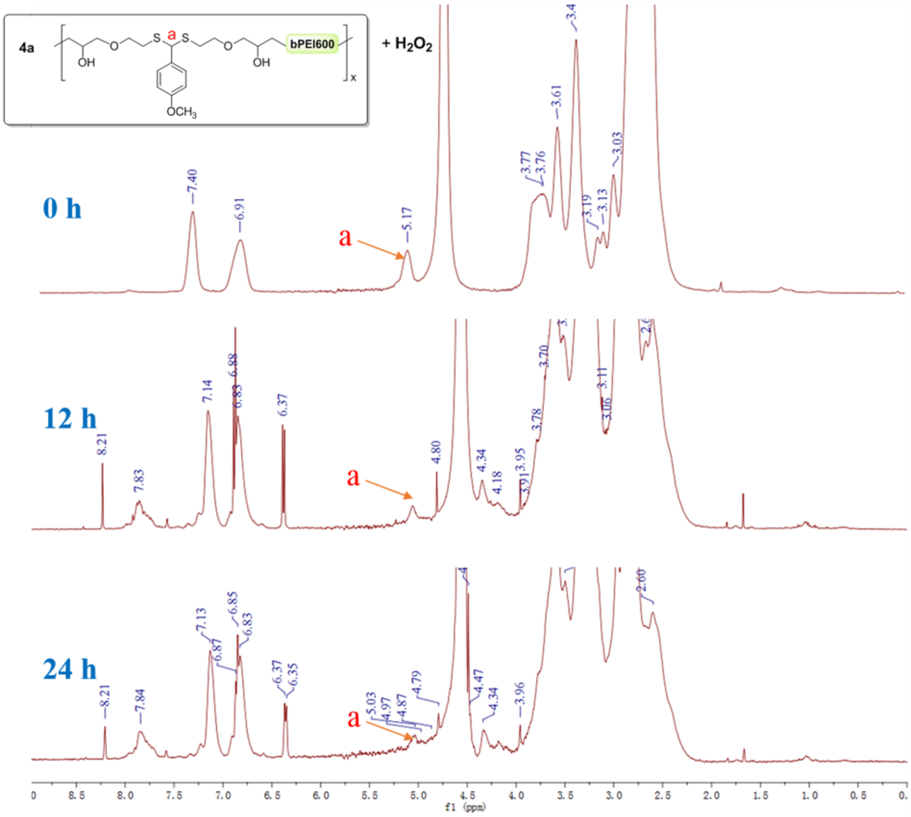

4a
bPEI600
+ H2O2
0 h
12 h
24 h
a
f1 (ppm)

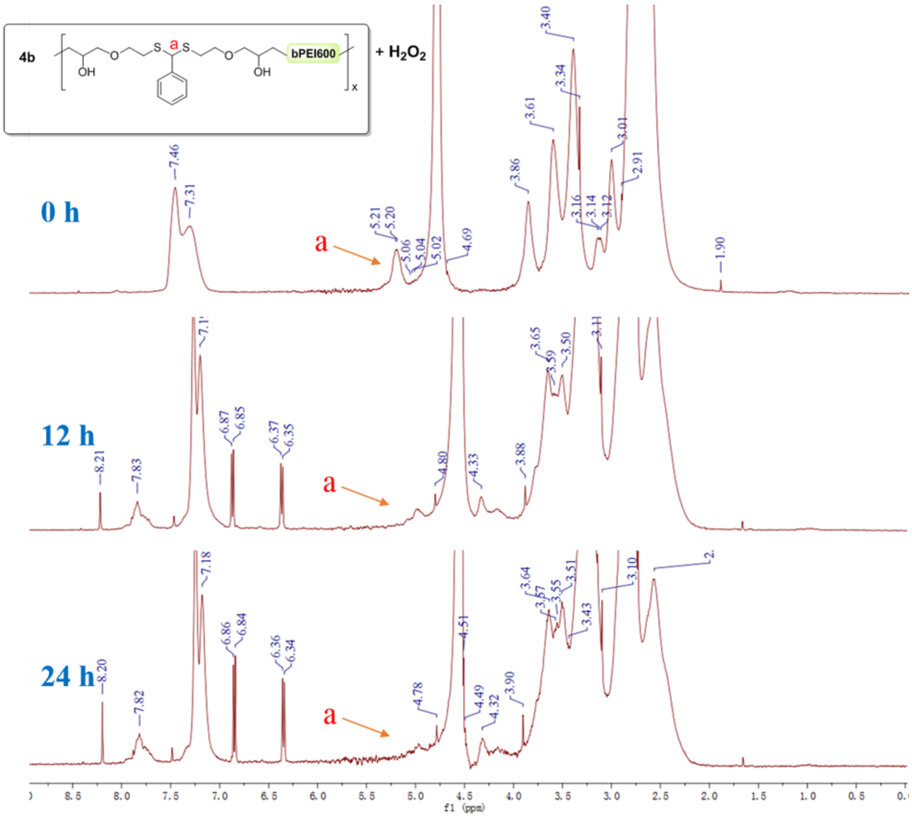

4b
bPEI600
+ H2O2
0 h
12 h
24 h
a
f1 (ppm)

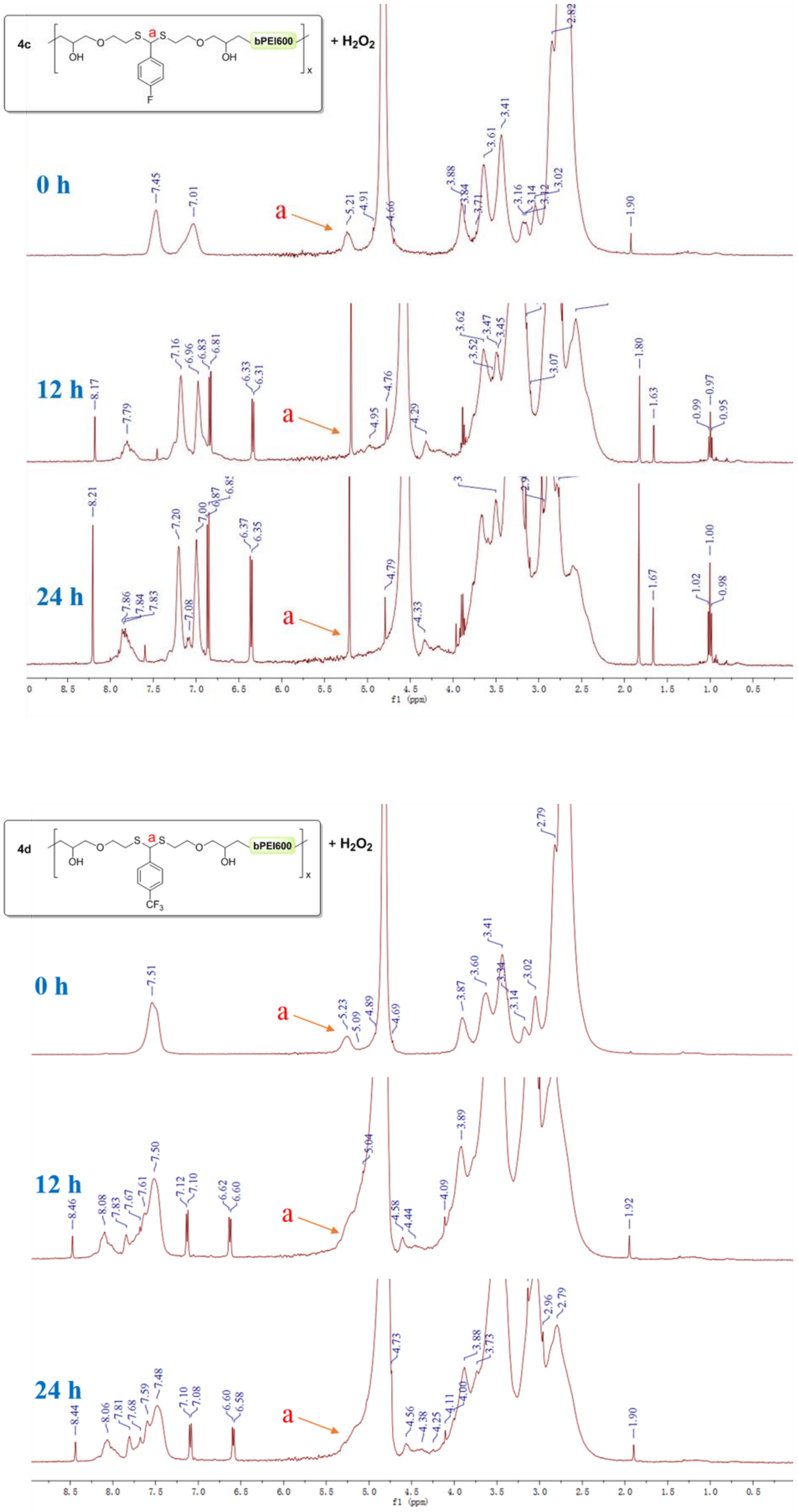

Fig. S2 Study of polymer **4** decomposition upon treatment with 100 mM H_2O_2 (simulated ROS conditions) for 0, 12 and 24 h using ^{1}H-NMR analysis.

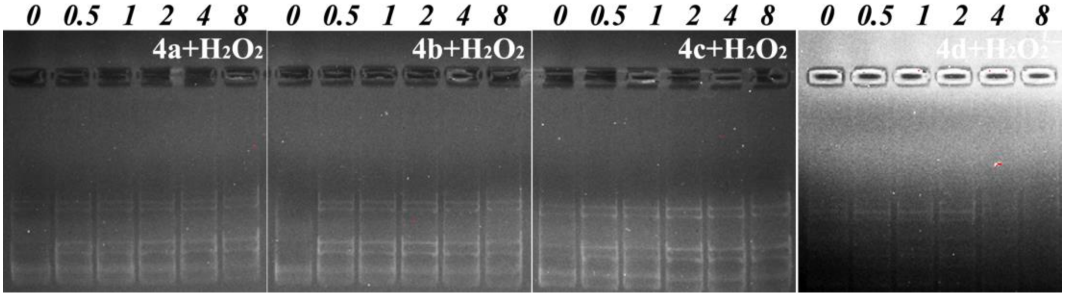

Fig. S3 Agarose gel electrophoresis of polymer/DNA polyplexes prepared at different w/w ratios after incubation with 100 mM H_2O_2 for 48 h at 37 °C. In each image, the first lane is the DNA control.

Table S1 Polymer/DNA ratio in terms of both weight ratio (w/w) and N/P ratio (polymer nitrogen/DNA phosphate) were used.

w/w ratio / N/P ratio / polymer	1.4	2	4	6
**4a**		8.74	17.49	26.22
**4b**		9.02	18.04	27.06
**4c**		8.85	17.71	26.55
**4d**		8.42	16.84	25.26
**PEI 25K**	10			
**PEI 600**			28.7	

Spectra

^{19}F-NMR Spectra

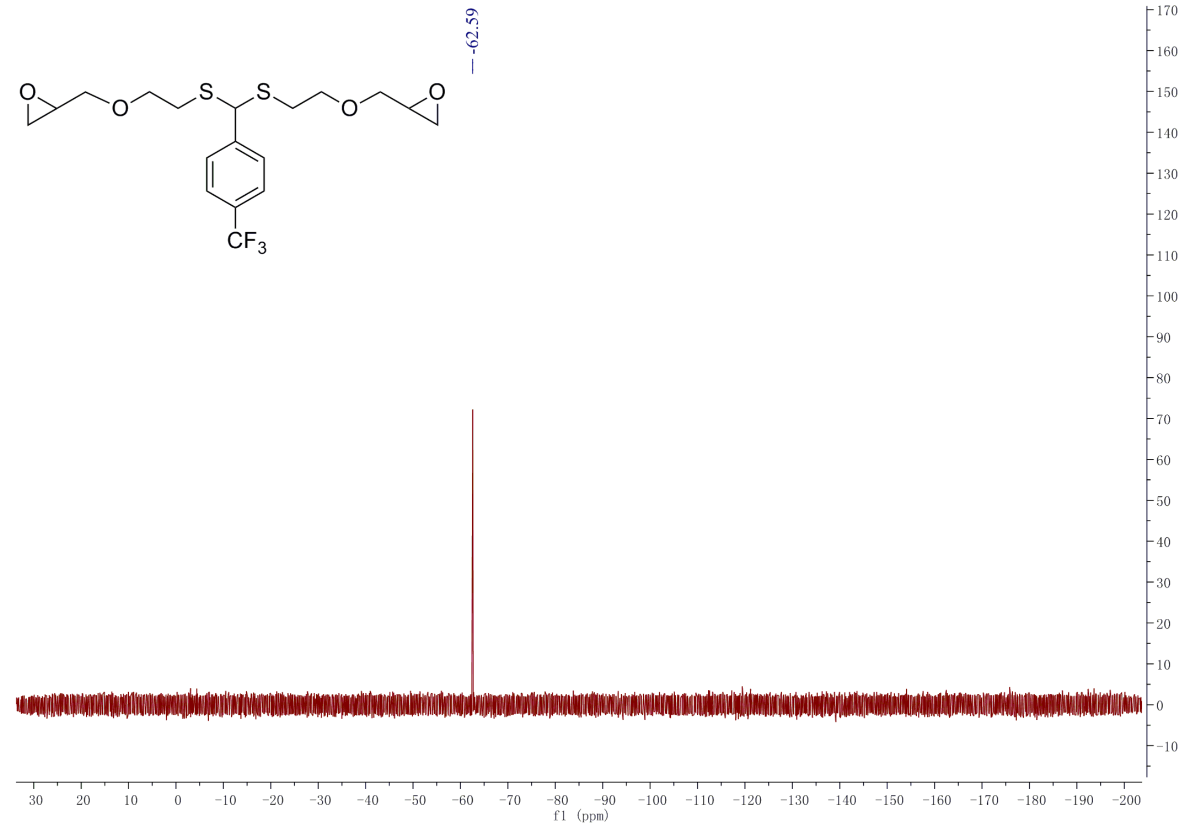

^{1}H-NMR Spectra

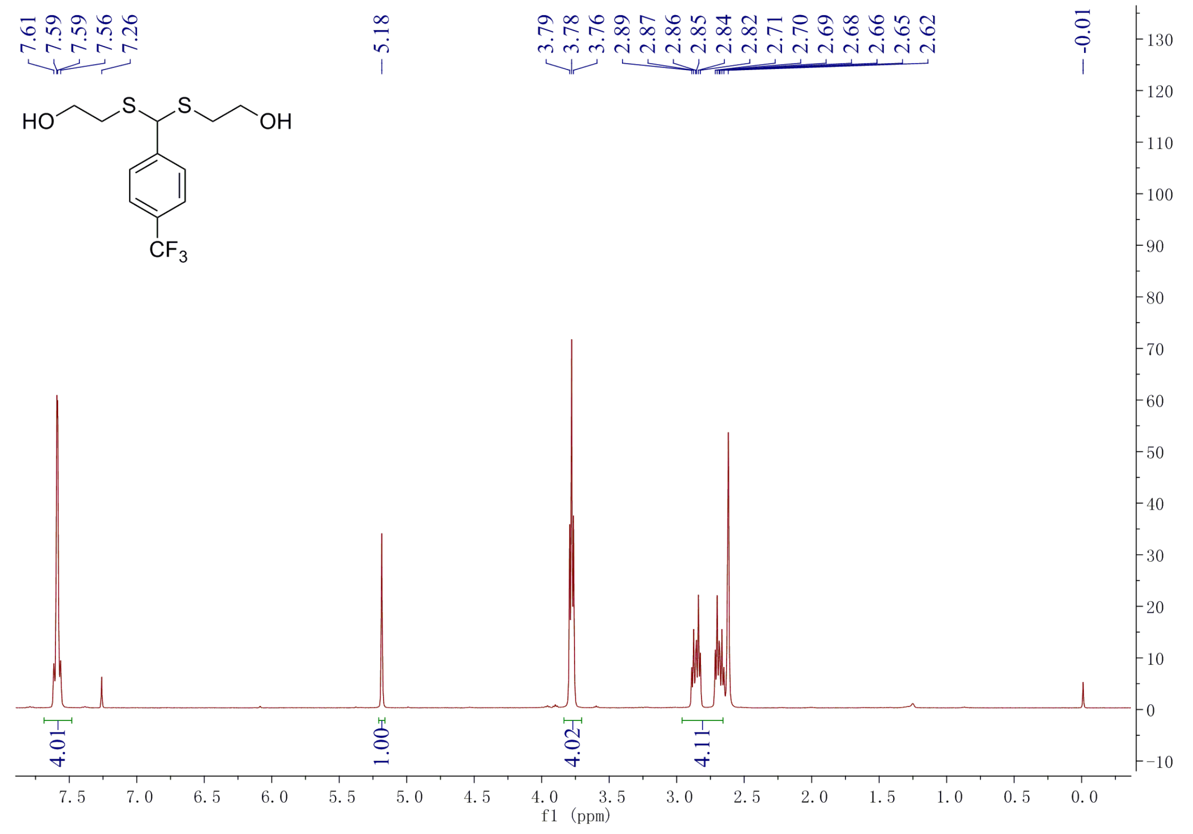

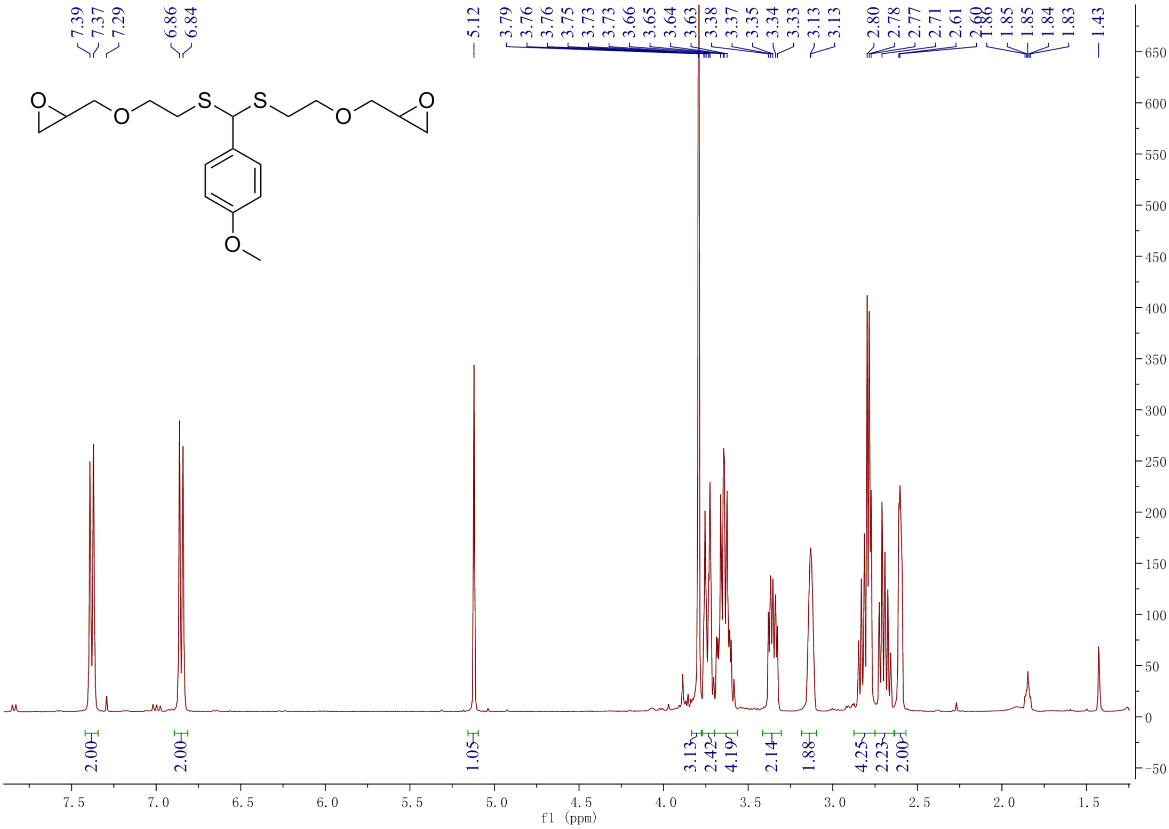
7.39 7.37 7.29 6.86 6.84 5.12 3.79 3.76 3.76 3.75 3.73 3.73 3.66 3.65 3.64 3.63 3.38 3.37 3.35 3.34 3.33 3.13 3.13 2.80 2.78 2.77 2.71 2.61 2.60 1.86 1.85 1.84 1.83 1.43
2.00 2.00 1.05 3.13 2.42 4.19 2.14 1.88 4.25 2.23 2.00
f1 (ppm)

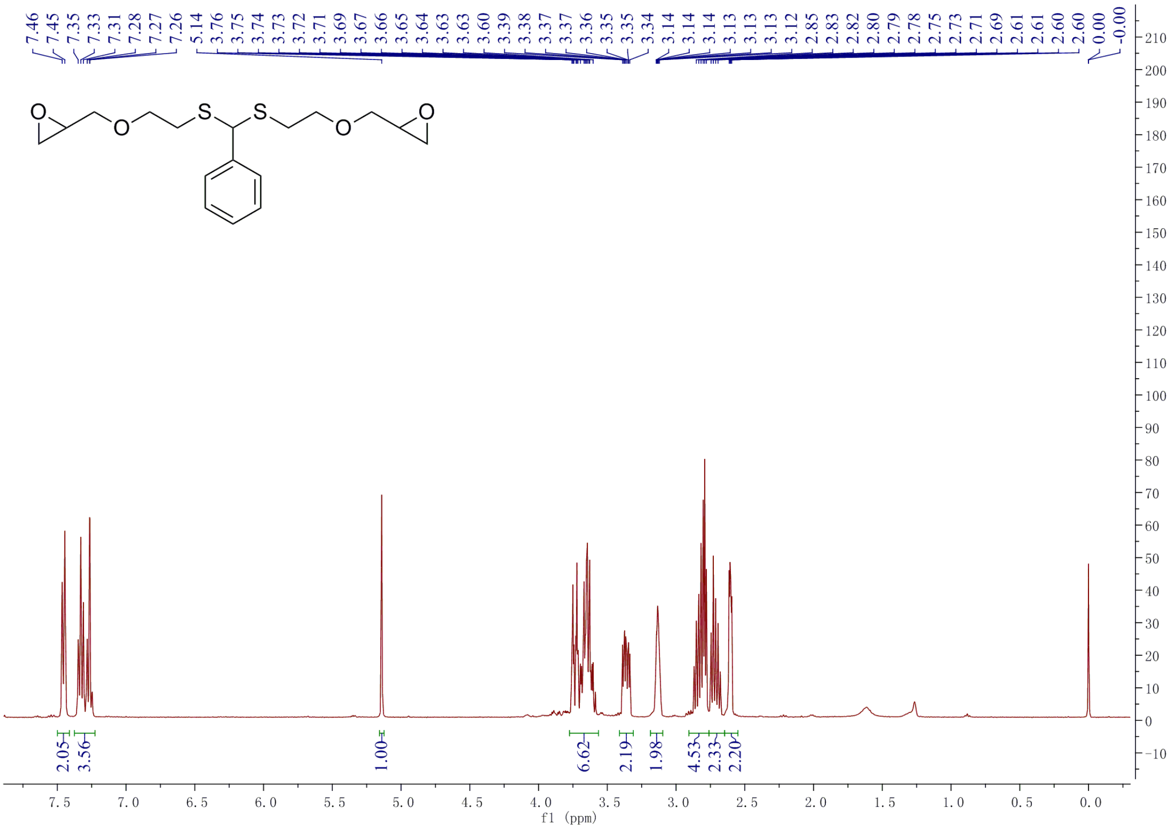
7.46 7.45 7.35 7.33 7.31 7.28 7.27 7.26 5.14 3.76 3.75 3.74 3.73 3.72 3.71 3.69 3.67 3.66 3.65 3.64 3.63 3.63 3.60 3.39 3.38 3.37 3.37 3.36 3.35 3.35 3.34 3.14 3.14 3.14 3.13 3.13 3.13 3.12 2.85 2.83 2.82 2.80 2.79 2.78 2.75 2.73 2.71 2.69 2.61 2.61 2.60 0.00 -0.00
2.05 3.56 1.00 6.62 2.19 1.98 4.53 2.33 2.20
f1 (ppm)

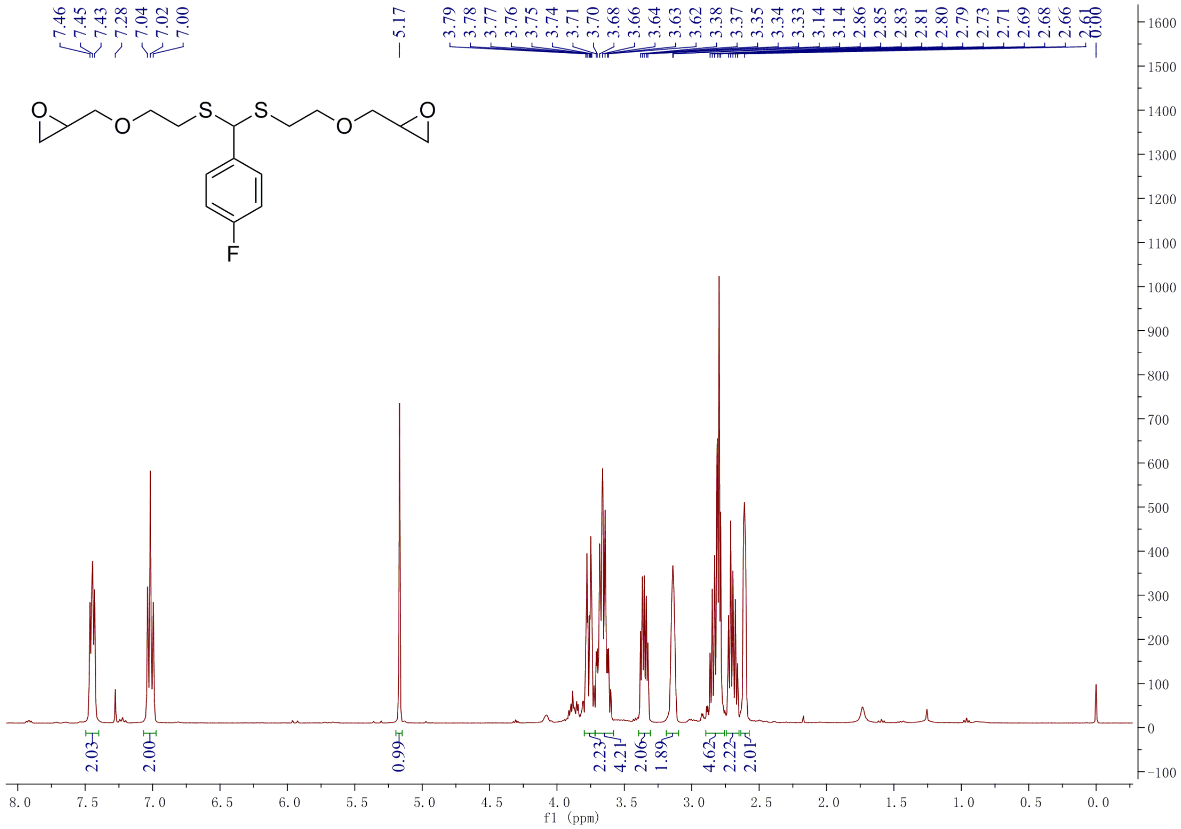

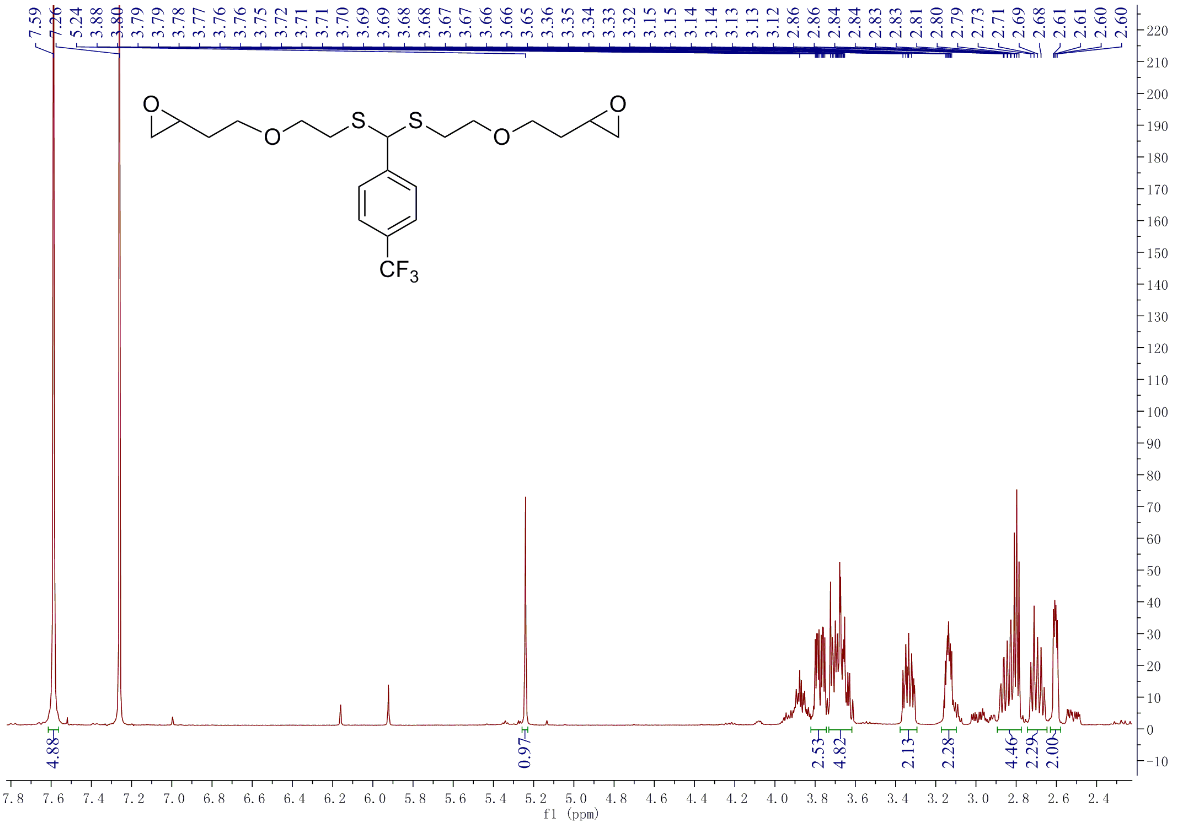

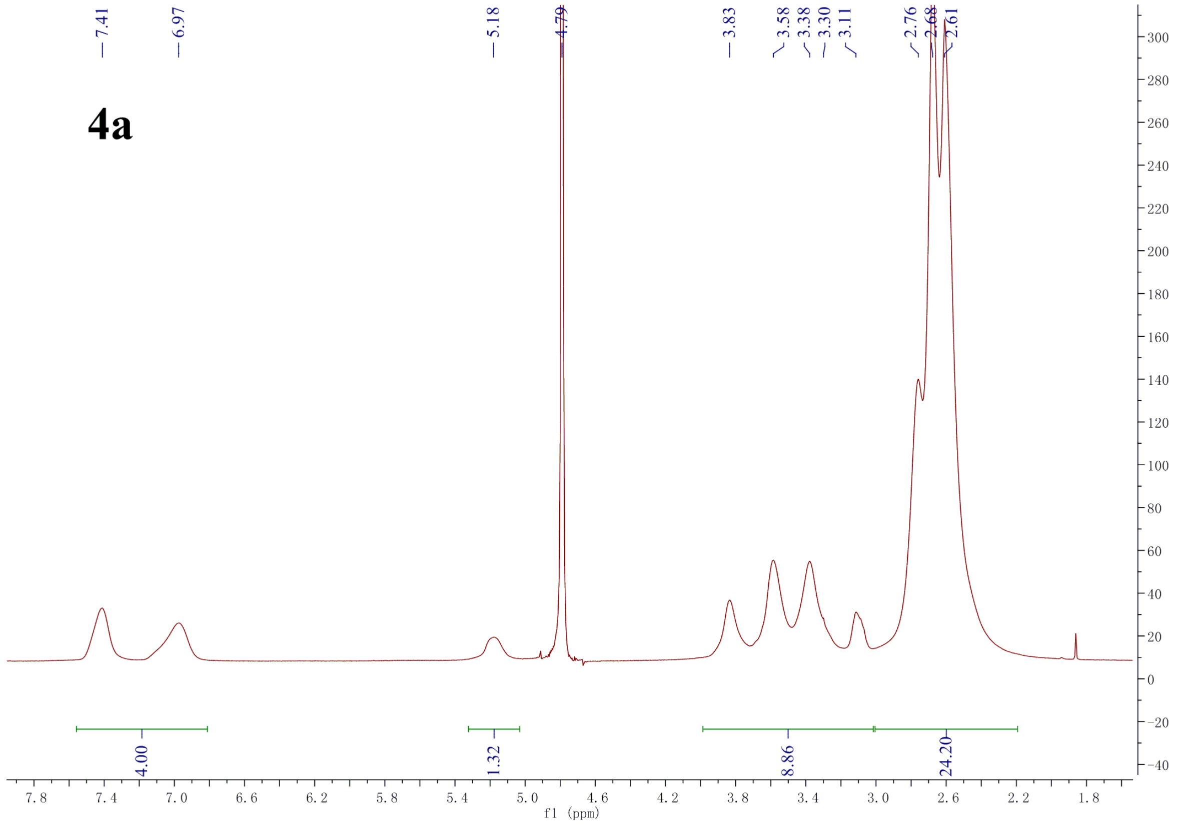
4a
7.41
6.97
5.18
4.79
3.83
3.58
3.38
3.30
3.11
2.76
2.68
2.61
4.00
1.32
8.86
24.20
f1 (ppm)

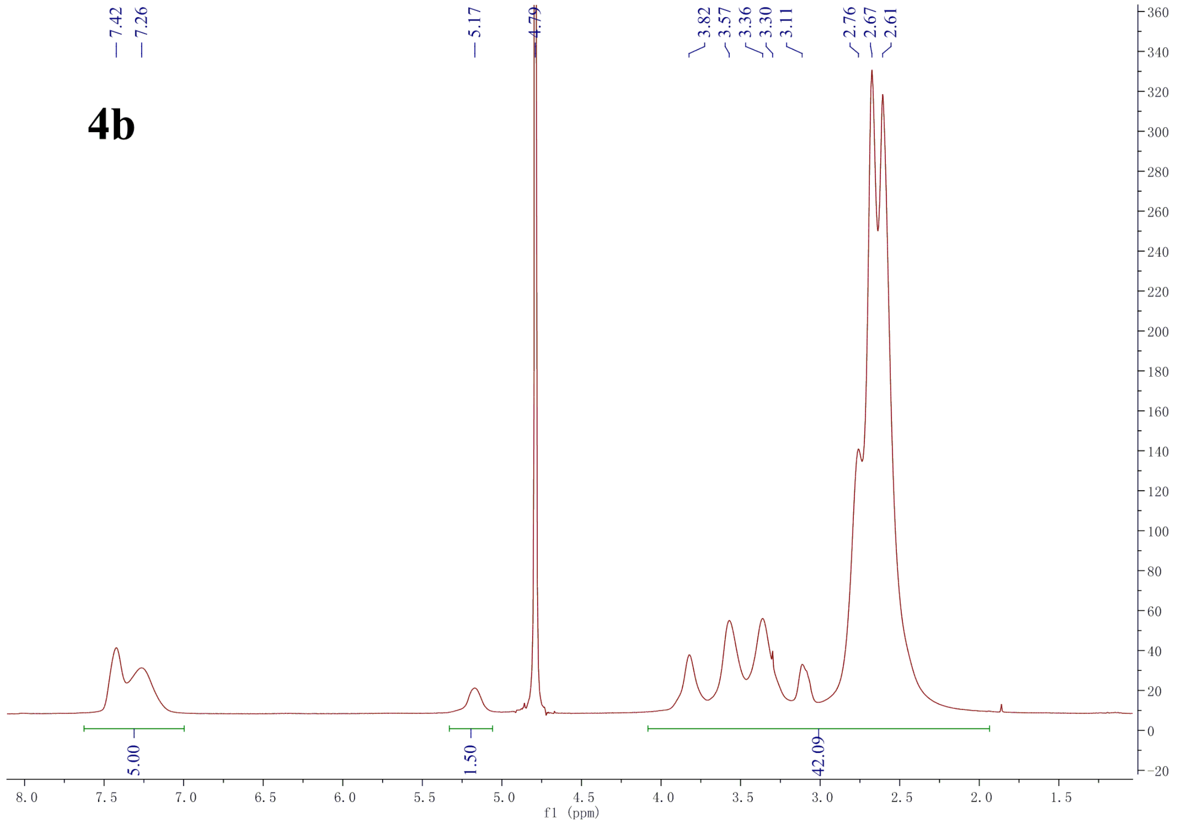
4b
7.42
7.26
5.17
4.79
3.82
3.57
3.36
3.30
3.11
2.76
2.67
2.61
5.00
1.50
42.09
f1 (ppm)

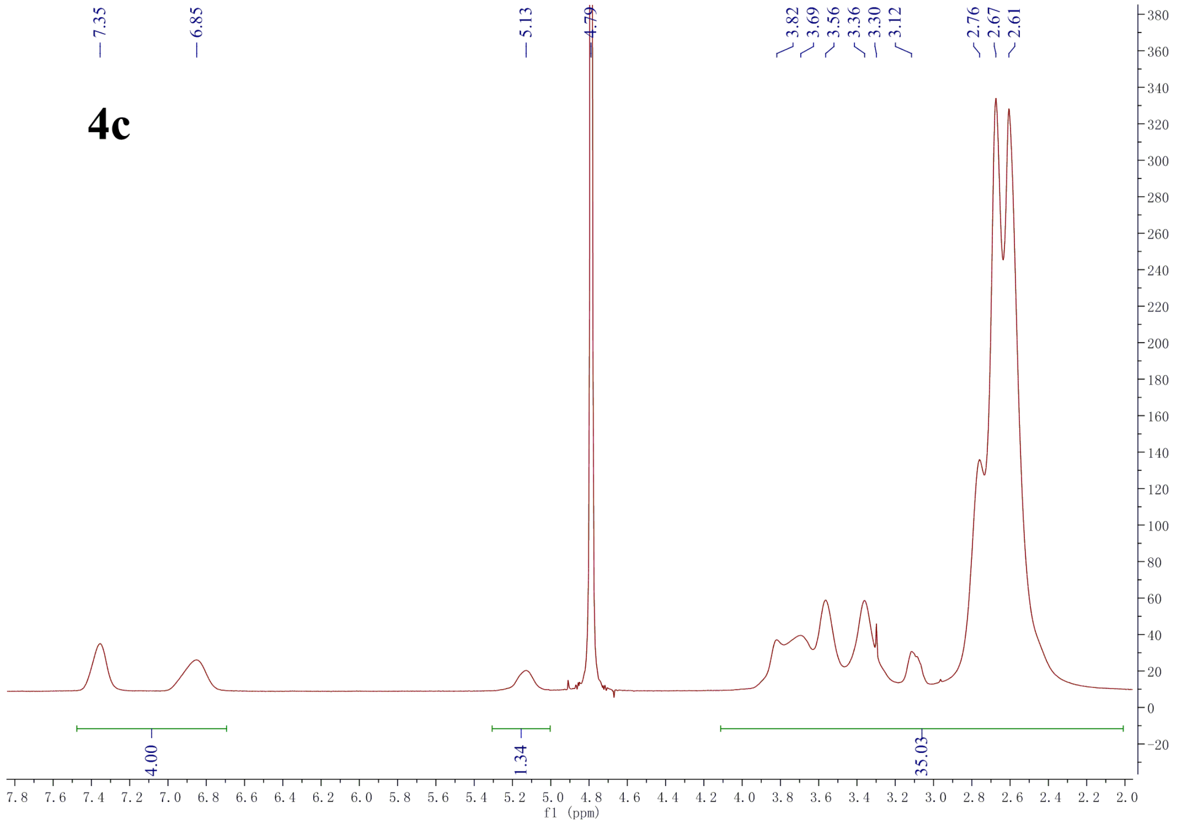

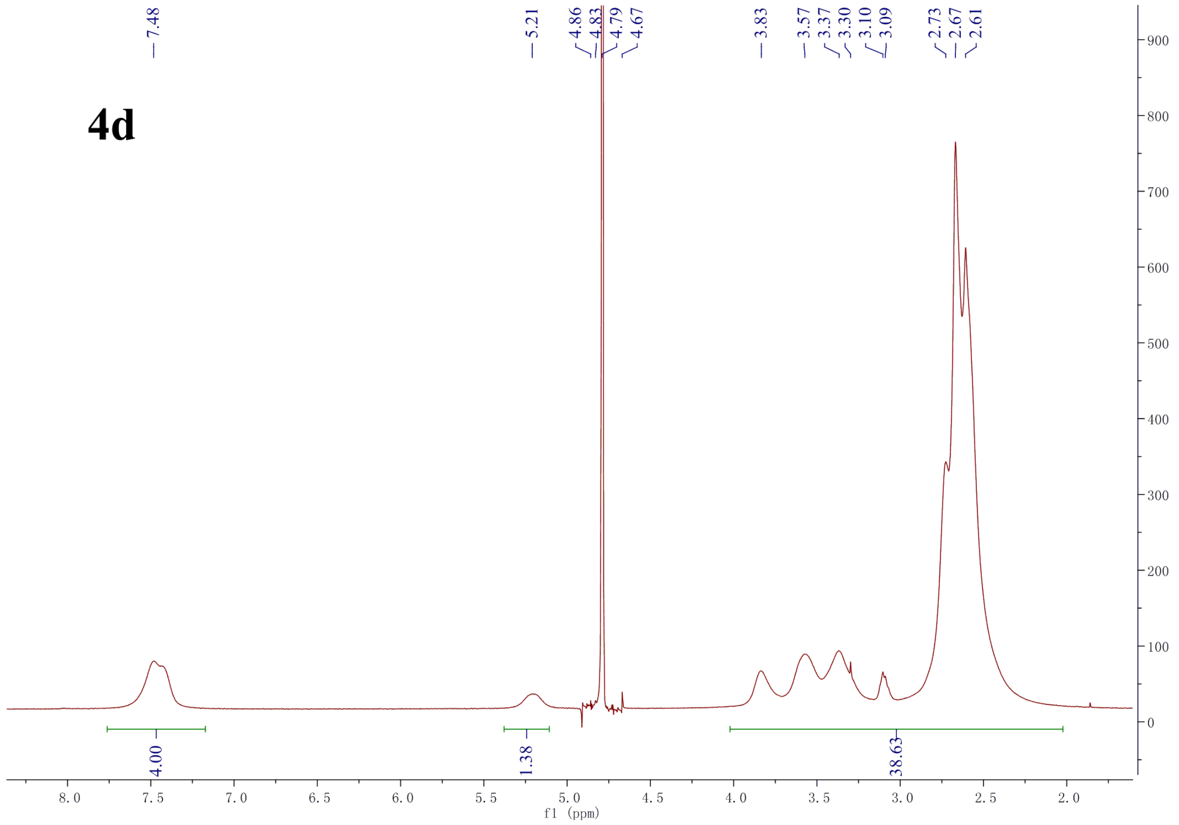

^{13}C-NMR Spectra

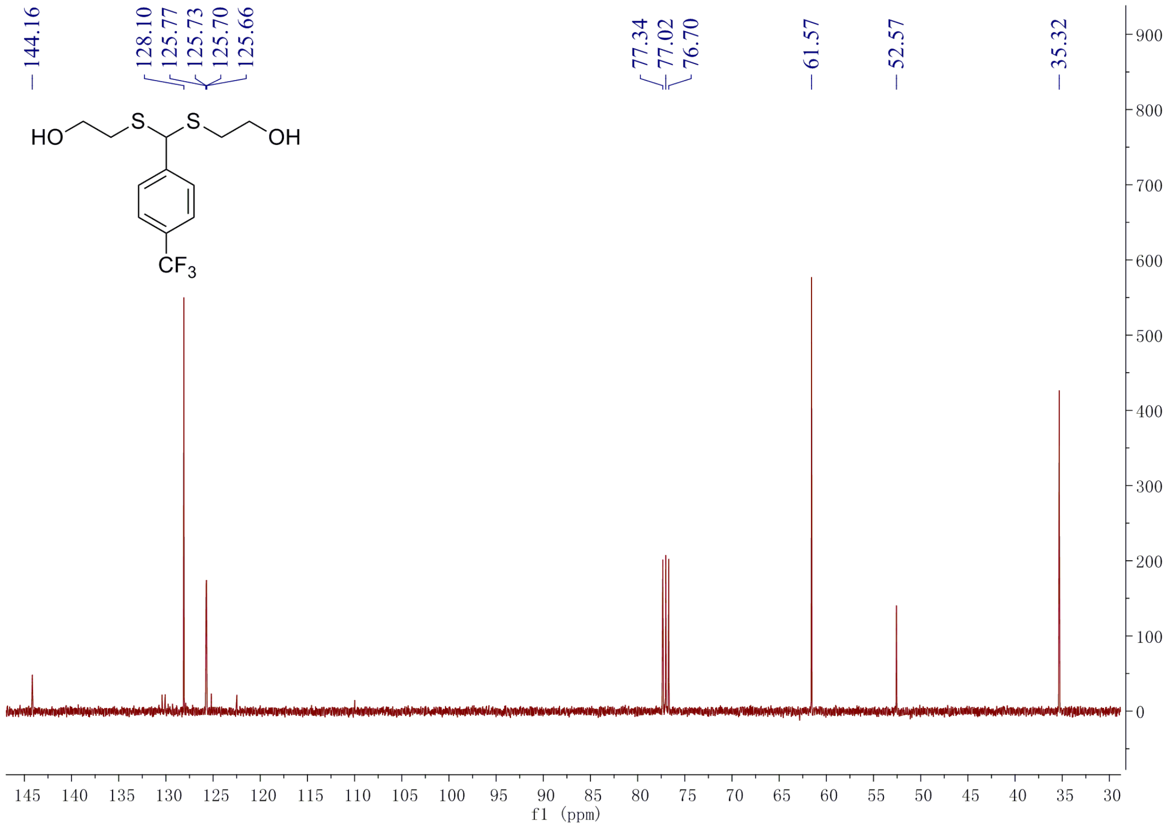
144.16
128.10
125.77
125.73
125.70
125.66
77.34
77.02
76.70
61.57
52.57
35.32
HO
S
S
OH
CF3
f1 (ppm)

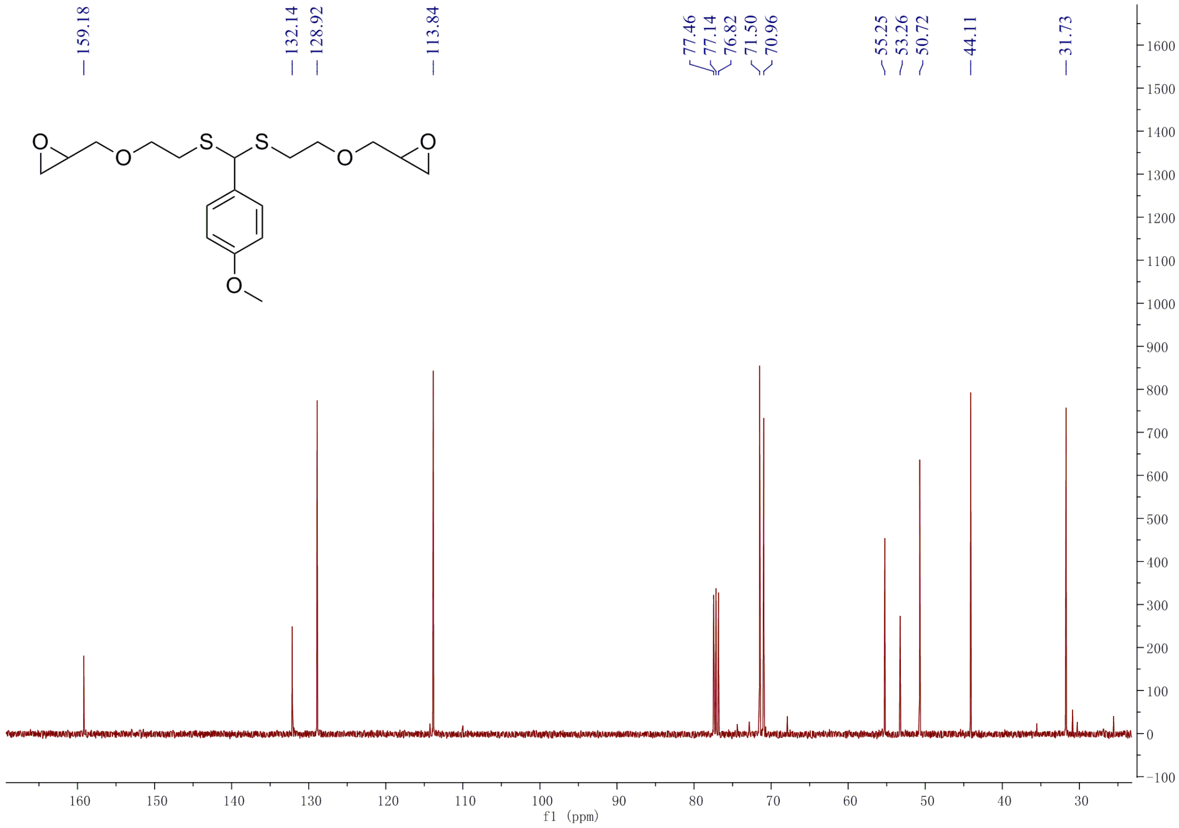
159.18
132.14
128.92
113.84
77.46
77.14
76.82
71.50
70.96
55.25
53.26
50.72
44.11
31.73
O
O
S
S
O
O
O
f1 (ppm)

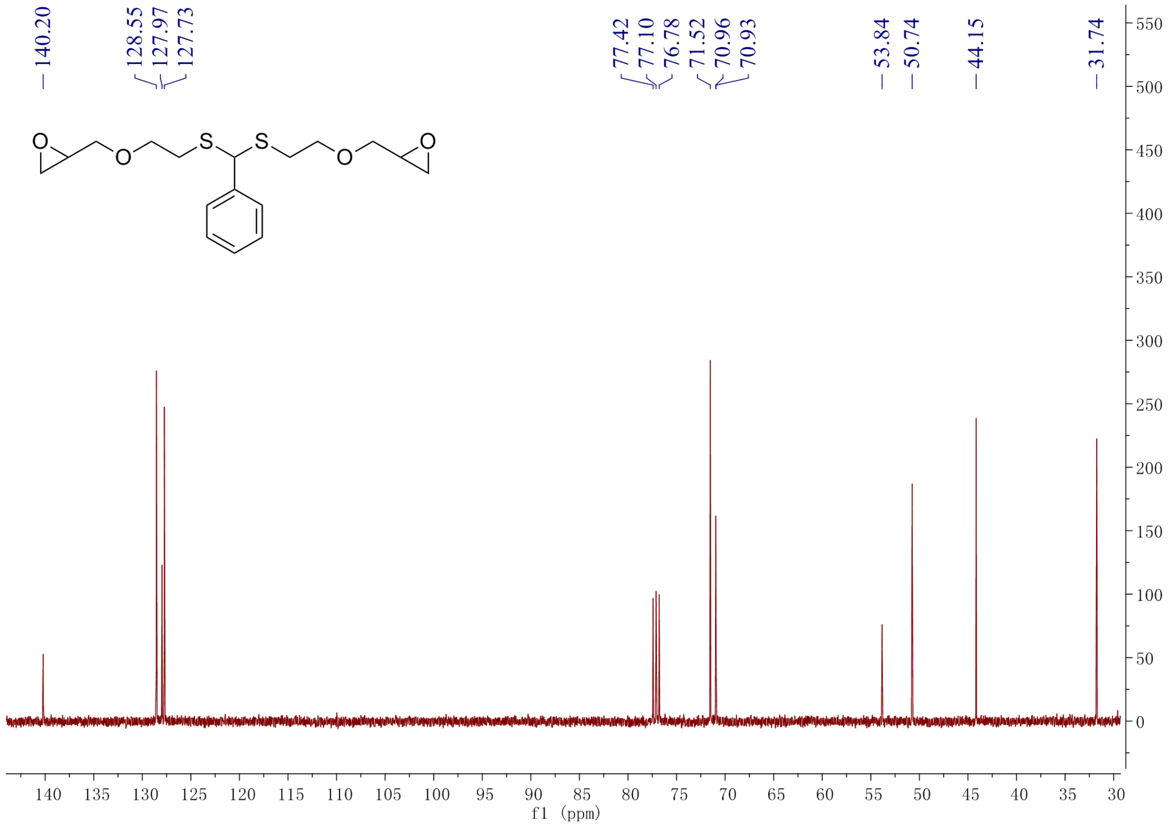
140.20
128.55
127.97
127.73
77.42
77.10
76.78
71.52
70.96
70.93
53.84
50.74
44.15
31.74
f1 (ppm)

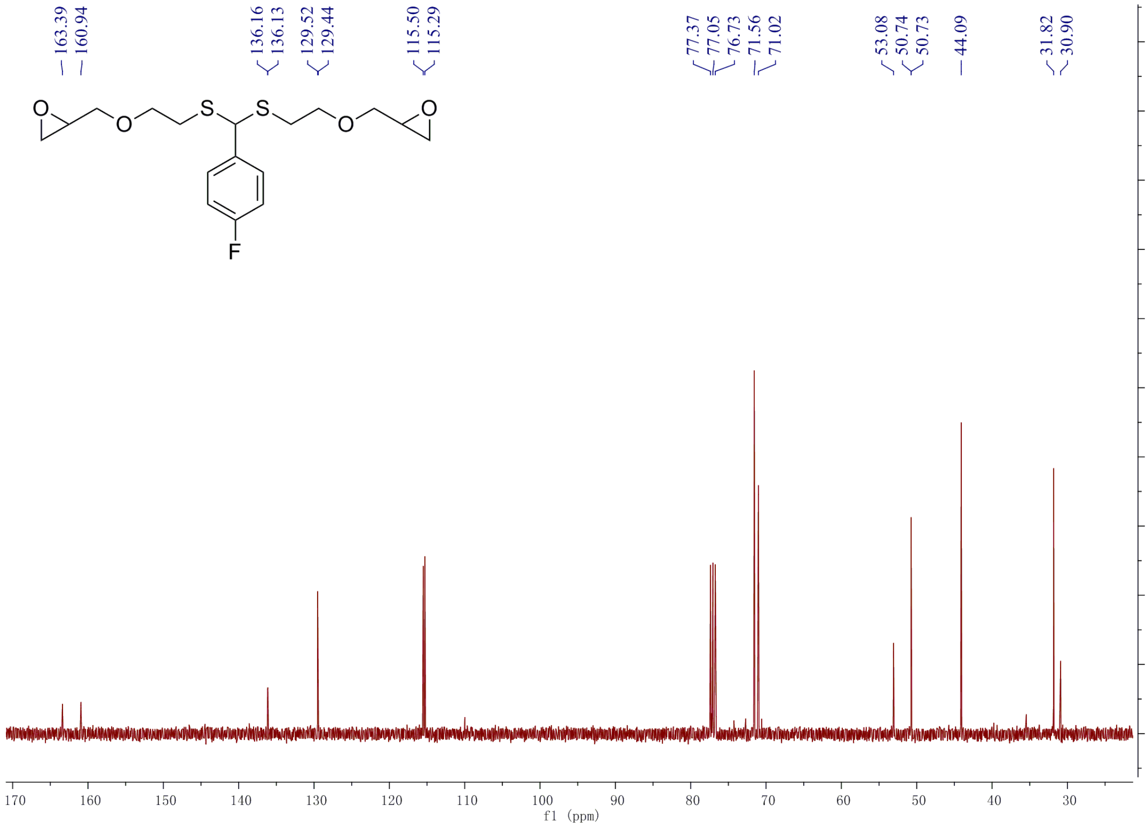
163.39
160.94
136.16
136.13
129.52
129.44
115.50
115.29
77.37
77.05
76.73
71.56
71.02
53.08
50.74
50.73
44.09
31.82
30.90
F
f1 (ppm)

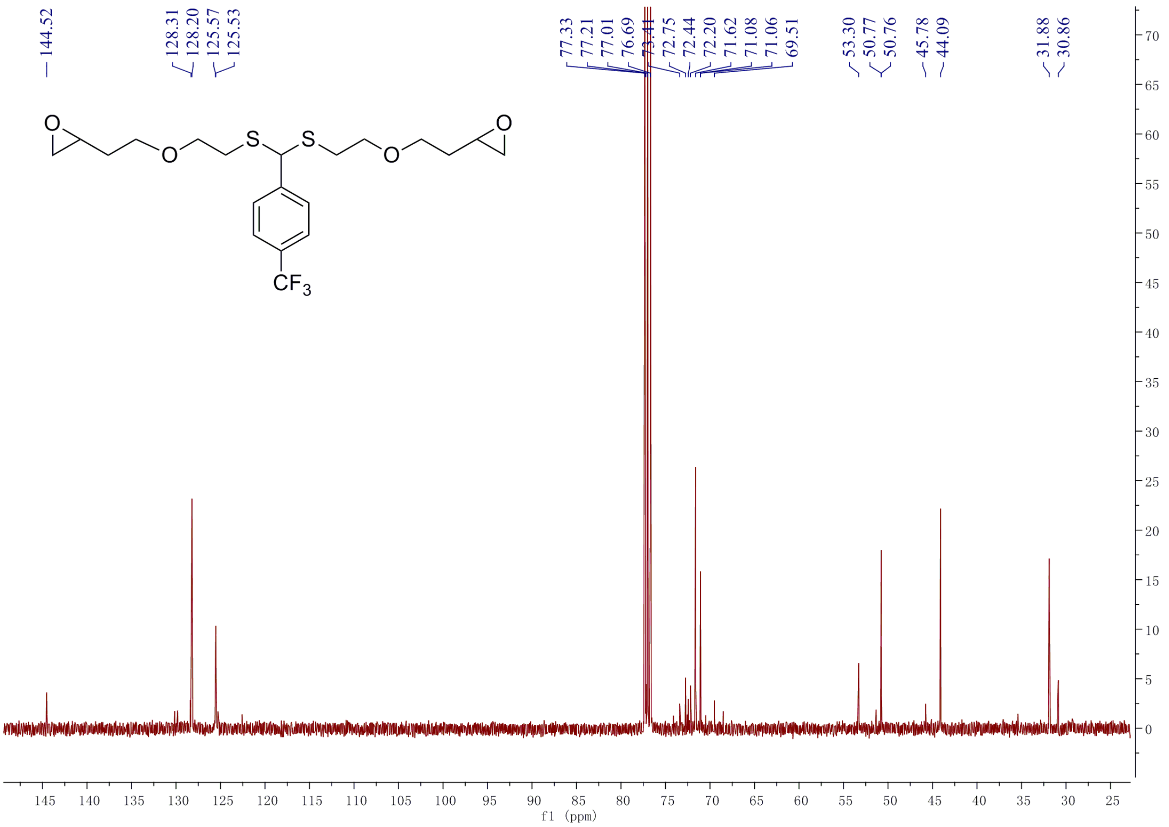

GPC

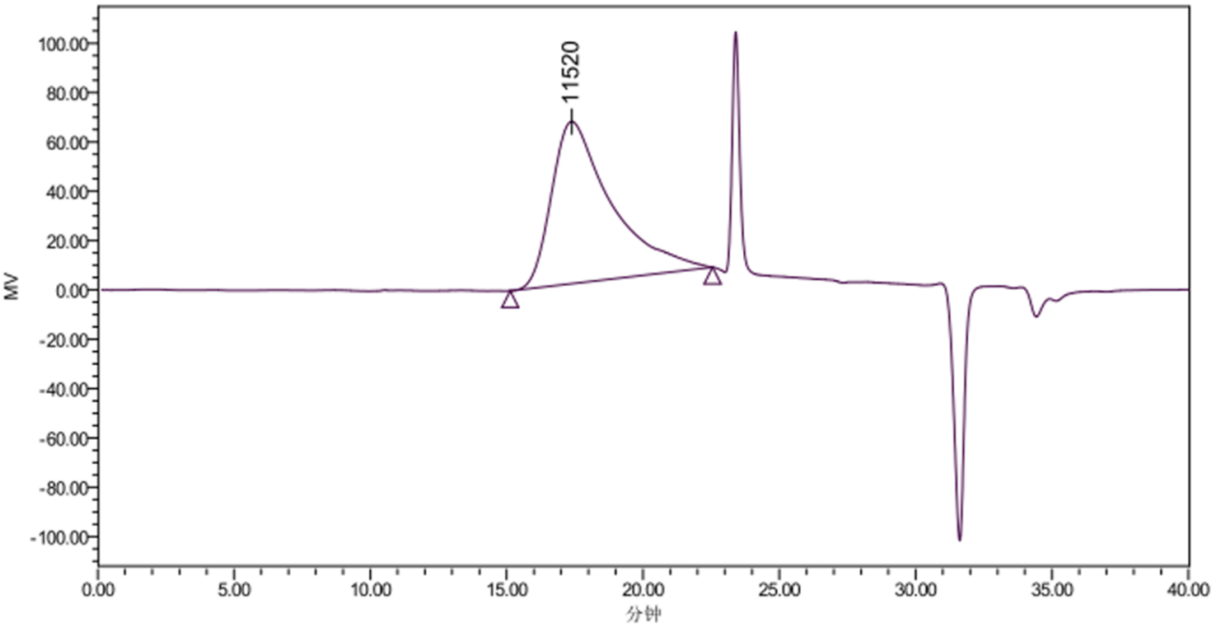

宽分布未知样相对峰表

	分布名	Mn (道尔顿)	Mw (道尔顿)	MP	Mz (道尔顿)	Mz+1 (道尔顿)	多分散性	Mz/Mw	Mz+1/Mw
1		5349	9871	11520	14803	19572	1.845554	1.499636	1.982787

4a

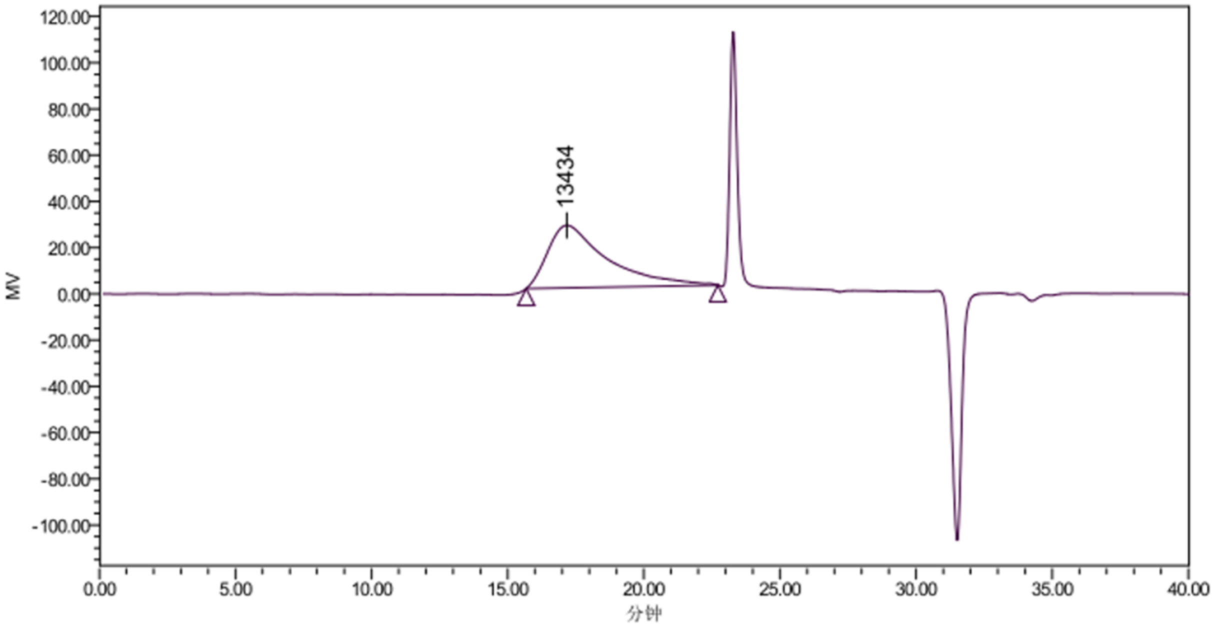

宽分布未知样相对峰表

	分布名	Mn (道尔顿)	Mw (道尔顿)	MP	Mz (道尔顿)	Mz+1 (道尔顿)	多分散性	Mz/Mw	Mz+1/Mw
1		5547	10916	13434	16168	20475	1.968078	1.481086	1.875709

4b

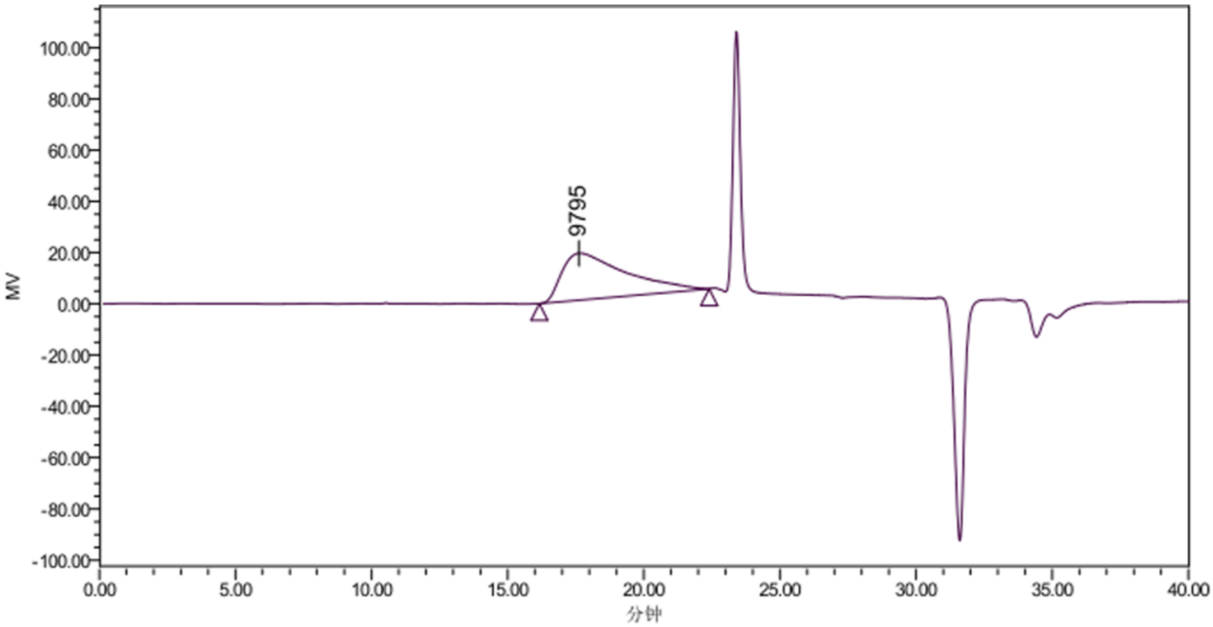

宽分布未知样相对峰表

	分布名	Mn (道尔顿)	Mw (道尔顿)	MP	Mz (道尔顿)	Mz+1 (道尔顿)	多分散性	Mz/Mw	Mz+1/Mw
1		4199	7123	9795	10114	12458	1.696495	1.419886	1.748911

4c

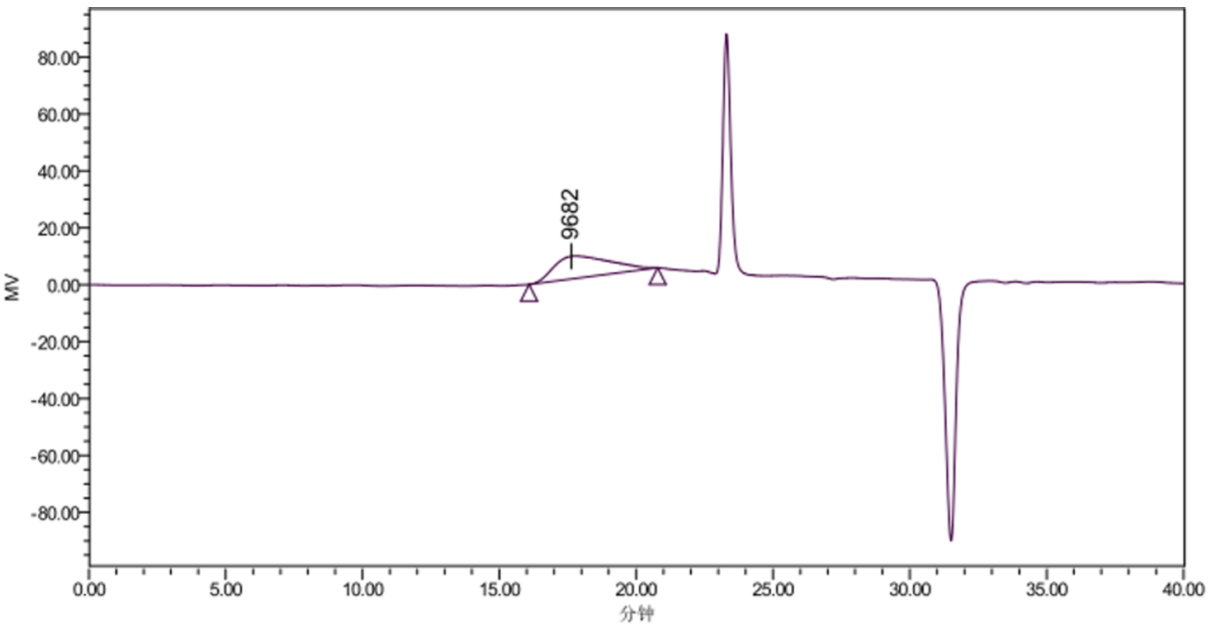

宽分布未知样相对峰表

	分布名	Mn (道尔顿)	Mw (道尔顿)	MP	Mz (道尔顿)	Mz+1 (道尔顿)	多分散性	Mz/Mw	Mz+1/Mw
1		5879	8209	9682	10781	13143	1.396357	1.313374	1.601084

4d
